# Protective Effect of Melatonin for Renal Ischemia-Reperfusion Injury: A Systematic Review and Meta-Analysis

**DOI:** 10.3389/fphys.2021.791036

**Published:** 2022-01-13

**Authors:** Rong-liang Dun, Tian-ying Lan, Jennifer Tsai, Jian-min Mao, Yi-qun Shao, Xiao-hua Hu, Wen-jing Zhu, Guang-chong Qi, Yu Peng

**Affiliations:** ^1^Urology Surgery, Yueyang Hospital of Integrated Traditional Chinese and Western Medicine Hospital, Shanghai University of Traditional Chinese Medicine, Shanghai, China; ^2^Nephrology, Shuguang Hospital, Shanghai University of Traditional Chinese Medicine, Shanghai, China; ^3^Urology Surgery, Shanghai Seventh People's Hospital, Shanghai, China

**Keywords:** renal ischemia-reperfusion injury, melatonin, systematic review, reactive oxygen species, meta-analysis

## Abstract

**Background:** Renal ischemia-reperfusion (I/R) injury is one of the major causes related to acute kidney damage. Melatonin has been shown as a powerful antioxidant, with many animal experiments have been designed to evaluate the therapeutic effect of it to renal I/R injury.

**Objectives:** This systematic review aimed to assess the therapeutic effect of melatonin for renal I/R injury in animal models.

**Methods and Results:** The PubMed, Web of Science, Embase, and Science Direct were searched for animal experiments applying melatonin to treat renal I/R injury to February 2021. Thirty-one studies were included. The pooled analysis showed a greater reduction of blood urea nitrogen (BUN) (21 studies, weighted mean difference (WMD) = −30.00 [−42.09 to −17.91], *p* < 0.00001), and serum creatinine (SCr) (20 studies, WMD = −0.91 [−1.17 to −0.66], *p* < 0.00001) treated with melatonin. Subgroup analysis suggested that multiple administration could reduce the BUN compared with control. Malondialdehyde and myeloperoxidase were significantly reduced, meanwhile, melatonin significantly improved the activity of glutathione, as well as superoxide dismutase. The possible mechanism for melatonin to treat renal I/R injury is inhibiting endoplasmic reticulum stress, apoptosis, inflammation, autophagy, and fibrillation in AKI to chronic kidney disease.

**Conclusions:** From the available data of small animal studies, this systematic review demonstrated that melatonin could improve renal function and antioxidative effects to cure renal I/R injury through, then multiple administration of melatonin might be more appropriate. Nonetheless, extensive basic experiments are need to study the mechanism of melatonin, then well-designed randomized controlled trials to explore the protective effect of melatonin.

## Introduction

Acute kidney injury (AKI) is defined as a sequence of clinical syndromes, refers to the sudden and continuous damage of renal function, which is manifested by a sharp reduction of glomerular filtration rate, with decreased urine output and azotemia (Levey and James, 2017). It is becoming increasingly clear that AKI is a non-negligible factor from chronic kidney disease (CKD) to chronic renal failure (Chawla and Kimmel, 2012). It was reported that 35 and 71% of AKI patients had incomplete recovery of renal function (Basile et al., 2001). Renal transplantation, systemic hypotension, hypovolemic shock, cardiovascular surgery, and partial nephrectomy as well as many other clinical conditions can lead to renal ischemia-reperfusion (I/R). As the most common cause related to AKI, renal I/R injury is an important factor leading to high mortality in intensive care units (Bansal et al., 2018; Smith et al., 2019). It would also cause the increase of antibodies, and the increased antibodies may impair renal transplantation, so as to explain the pathophysiological relationship between delayed renal allograft function and rejection in renal transplant recipients (Fuquay et al., 2013).

In recent years, researchers have deeply explored the changing signal pathways related to I/R, then put forward various mechanisms to explain the renal tissue damage. A reduced blood supply can lead to a low energy condition, causing loss of cell polarity and disorganization of organelles, especially mitochondrion, in the ischemia stage of renal I/R injury. Then subsequent reperfusion induces cause accumulation of a large number of reactive oxygen species (ROS) leading to oxidative stress, as well as mitochondrial and microvascular dysfunction, leading to more ROS production. ROS is generally considered as a key factor in renal I/R-induced injury. The release and accumulation of ROS activate a series of signaling pathways, including inflammation, apoptosis, autophagy, and endoplasmic reticulum (ER) stress in kidney (Inagi, 2009). The formation of the fibrotic tissues is the final manifestation from AKI to CKD situation, such as glomerulosclerosis, tubular atrophy, as well as renal interstitial fibrosis, which are characterized by fibroblasts activation and macrophage infiltrates (Webster et al., 2017).

Melatonin is one of the hormones secreted by the pineal gland that can construct circadian rhythm to regulate sleep and enhance immune function (Patel et al., 2020). Because of its high lipophilicity, melatonin can easily reach the subcellular structure through the cell membrane (Reiter et al., 2001). Melatonin has been shown as an excellent antioxidant due to the powerful ability to reduce ROS (Zhang and Zhang, 2014). This characteristic offers melatonin the ability to protect DNA against oxidative stress. In addition, melatonin also shows strong anti apoptotic and anti-inflammatory effects and other significant cytoprotective effects (Carrascal et al., 2018; Mortezaee et al., 2019; Xia et al., 2019). At present, many animal experimental studies related to the therapeutic action of melatonin for renal I/R injury have been published, however, there is still no final conclusion and whether it is due to its antioxidant properties. Also, a randomized controlled trial using melatonin on renal I/R injury in transplant patients was published in 2019, as only 20 patients were enrolled in each group, thus there were still doubts about its effect (Panah et al., 2019).

Systematic review is a secondary study that attempts to collect all evidence, use methodological methods to minimize the risk of bias and better evaluate the treatment effect in combine with individual studies. Therefore, we systematically reviewed and meta-analyzed the therapeutic action of melatonin in renal I/R injury rat models, and the mechanism of the renal protective effect of melatonin was also discussed.

## Methods

### Search Methods

A systematic literature search was performed in PubMed, Web of Science, Embase, and Science Direct from their inception date to February 2021. Keywords included “ischemia/reperfusion injury,” “ischemia-reperfusion injury,” “I/R injury,” “melatonin,” “N-acetyl-5-methoxytryptamine,” and “MT,” “MLT.” All object types were limited to animals, and there are no restrictions on language.

### Study Selection

Two investigators (DRL and YM) screened the titles and abstracts of studies identified for this review independently. Download the full text of the screened articles to determine whether they meet the predetermined inclusion and exclusion criteria. Agreement of the study selection was determined using the quadratic-weighted kappa value (Kw). The result was accepted if the Kw value was >0.75. Disagreements were resolved through discussion with the third reviewers (YP).

### Eligibility Criteria

#### Types of Studies

The controlled studies that evaluating melatonin administrated *in vivo* to renal I/R injury in experimental animals were included. Only *in vitro* experiments, case reports, and case series studies were ruled out. The study had no restrictions on language, publication date, as well as publication status.

#### Types of Animals

The study included experimental animals of any species, week age, as well as gender to induce renal I/R injury. The time of ischemia and reperfusion was not limited. Kidney transplantation, folic acid, and genetically modified induced AKI models were excluded.

#### Types of Intervention

Any types of melatonin that were compared to empty control or placebo were included. Dosage, dosage form, dissolvent, administration route, as well as melatonin administration time or duration were not limited. Placebo controls were not limited to physiological saline, absolute ethanol, or DMSO.

#### Type of Outcome

Serum creatinine (SCr) as well as blood urea nitrogen (BUN) that evaluated the renal function were considered as primary outcomes, ignoring the different detection methods. The biochemical indexes related to oxidative stress were secondary outcomes, including superoxide dismutase (SOD), malondialdehyde (MDA), myeloperoxidase (MPO), as well as glutathione (GSH). These tests were the most frequently used indicators of oxidative stress. The detection methods of MDA, MPO, SOD, and GSH and their corresponding numerical units are not limited.

### Data Extraction

Data was extracted from the included studies independently by two reviewers (DRL and LTY), agreement of the data was determined using the quadratic-weighted kappa value (Kw). The result was accepted if the Kw value was >0.75. Disagreements were resolved through discussion with the third reviewers (YP). The following relevant information of each study was extracted in Table 1: (1) study's characteristics (i.e., first author's name, publication year, country), (2) animals (i.e., number of included animals, strain, gender, weight/week age), (3) I/R injury (i.e., kidney excised or not, duration of ischemia, left or right, unilateral or bilateral), (4) interventions (i.e., group, dosage and time, administration route, timing, and duration), and (5) data about BUN, SCr, MDA, MPO, SOD, and GSH. The mean results, standard deviation (SD), as well as the sample size of animals in each group were collected.

**Table 1:** Characteristics of the studies included in the review.

**References**	**Country**	**Animals**	**I/R**	**No. of Animals**	**Groups**	**Met**	**Renal function**
Sener et al. (2002)	Turkey	Male Wistar albino rats (200–250 g)	Right excised for 2 wk, left ischemia for 45 min, followed by reperfusion for 1, 3, 6, 24, 48 h, or 1 wk	6/6/6/6/6	A. Control B. I C. I-Mel D. I/R1/3/6/24/48 h/1 wk E. I/R1/3/6/24/48 h/1 wk-Mel	10 mg/kg, *s.c*., 15 min before ischemia and reperfusion period	BUN, Scr
Kunduzova et al. (2003)	France	Male SD rats weighing 200–250 g	Right ischemia for 45 min, followed by reperfusion for 6 h	4-6/4-6/4-6/4-6	A. Sham B. Sham + Mel C. I/R + vehicle D. I/R + Mel	5 mg/kg, *i.v*. 15 min before ischemia	BUN, Scr, Scoring of tubular necrosis
Sahna et al. (2003)	Turkey	Male Wistar rats (150–200 g)	Right excised, left ischemia for 60 min, followed by reperfusion for 24 h	8/8/8/8	A. Sham B. I/R C. I/R + Mel (before ischemia) D. I/R + Mel (before reperfusion)	4 mg/kg, *i.p*., 10 min before ischemia	/
Rodríguez-Reynoso et al. (2004)	Mexico	Male SD rats (250–300 g)	Right excised, left ischemia for 75 min, followed by reperfusions	5/5/5/5	A. Sham B. I/R + vehicle C. I/R + Mel D. Sham + Mel	10 mg/kg, *i.p*., 15 min before ischemia	Scr
Aktoz et al. (2007)	Turkey	Male Wistar-albino rats (233–339 g)	Left ischemia for 60 min, followed by reperfusion for 60 min	6/6/6/6	A. Sham B. I/R C. I/R + Mel D. I/R + Vitamin E	10 mg/kg *i.p*., 72, 48, 24 h, and 30 min before ischemia	Scr, BUN
Kurcer et al. (2007a)	Turkey	Male SD rats (150–250 g)	Left ischemia for 30 min, followed by reperfusion for 24 h	8/5/10/7/10/8	A. Control B. Diabetic C. I/R D. Diabetic + I/R E. Mel + I/R F. Mel + diabetic + I/R	4 mg/kg/day, *i.p*. daily for 15 days prior to ischemia	Scr, BUN
Kurcer et al. (2007b)	Turkey	Male Wistar albino rats (150–250 g)	Right excised, left ischemia for 60 min, followed by reperfusion for 24 h	8/8/8/8/8/8	A. Control B. Mel + control C. Sham D. Mel + sham E. I/R F. Mel + I/R	10 mg/kg, *i.p*., 10 min prior to ischemia	Scr, BUN
Fadillioglu et al. (2008)	Turkey	Male SD rats (150–250g)	Left ischemia for 30 min, followed by reperfusion for 24 h	7/5/7/7/7/7	A. Control B. Diabetic C. I/R D. Diabetic + I/R E. I/R + Mel F. I/R + diabetic + Mel	4 mg/kg, *i.p*., during 15 days prior to ischemia	ALT, AST
Ersoz et al. (2009)	Turkey	Male SD rats (250–300 g)	Bilaterally ischemia for 60 min, followed by reperfusion	8/8/8/8	A. Sham B. I/R C. I/R + Mel D.I/R + 1400W	10 mg/kg, *i.p*., 60 min before ischemia	Scr, BUN, AST
Sinanoglu et al. (2012)	Turkey	Male Wistar rats (200–250 g)	Right excised, left ischemia for 45 min, followed by reperfusion for 45 min	6/6/6/6/6	A. Control B. I/R C. Mel + I/R D. Vitamin D3 + I/R E. Mel + Vitamin D3 + I/R	10 mg/kg, *i.p*. for 7 days, a week prior to I/R	Scr, BUN, ALT, AST
Ahmadiasl et al. (2013)	Iran	male Wistar Albino rats (weighing 200–300 g)	Right excised, left ischemia for 45 min, followed by reperfusion for 24 h	10/10/10/10	A. I/R + vehicle B. I/R + Mel C. I/R + erythropoietin D. I/R + Mel + erythropoietin	10 mg/kg *i.p*., 10 min before ischemia	BUN
Sezgin et al. (2013)	Turkey	Male Wistar rats (200–250g)	Right excised, left ischemia for 45 min, followed by reperfusion for 45 min	6/6/6/6/6	A. Control B. I/R D. I/R + Mel E. I/R + Vitamin D3 F. I/R +Vitamin D3 + Mel	10 mg/kg, *i.p*., for 7 days prior to ischemia	Scr, BUN
Ahmadiasl et al. (2014a)	Iran	Male Wistar-Albino rats (200–300 g)	Right excised, left ischemia for 45 min, followed by reperfusion for 24 h	10/10/10/10/10	A. Sham B. I/R C. I/R + Mel D. I/R + erythropoietin E. I/R + erythropoietin + Mel	10 mg/kg *i.p*., 10 min prior to ischemia.	BUN
Ahmadiasl et al. (2014b)	Iran	Male Wistar- Albino rats (200–300 g)	Right excised, left ischemia for 45 min, followed by reperfusion for 24 h	10/10/10/10	A. Sham B. I/R C. I/R + Mel D. I/R + erythropoietin	10 mg/kg, *i.p*., 10 min prior to ischemia	Scr
Cetin et al. (2014)	Turkey	Albino New Zealand male rabbits	Left ischemia for 1 h, followed by reperfusion for 3 h	6/6/6/6/6	A. Control B. I C. I/R D. I/V/R E. I/R + Mel	2.5 mg/kg *i.p*., 1 h prior to ischemia	Scr, BUN
Sehajpal et al. (2014)	India	Male wistar Rats (200–250 g)	Both ischemia for 40 min, followed by reperfusion for 24 h	8/8/8/8/8/8	A. Control B. Sham C. I/R D. I/R + Mel (4 mg/kg) E. I/R + Mel (10 mg/kg) F. I/R + Mel+mifepristone	4/10 mg/kg, *i.p*., 30 min prior to ischemia	BUN, Serum progesterone, CrCl, Uric acid, Serum potassium
Hadj Ayed Tka et al. (2015)	Tunisia	Male Wistar rats (200–250 g)	Both ischemia for 60 min, followed by reperfusion for 120 min	6/6/6	A. Sham B. I/R C. I/R + Mel	40 mg/kg *i.p*., 30 min prior to ischemia	Jablonski score, Scr clearance
Oguz et al. (2015)	Turkey	Male Wistar albino rats (180–300 g)	Right excised, left ischemia for 1 h, followed by reperfusion for 24 h	6/4/12/12	A. Control B. LPS C. I/R D. I/R + Mel	10 mg/kg *i.p*., before ischemia	Renal tubular injury
Yilmaz et al. (2015)	Turkey	Wistar albino type male rats (250–260 g)	Left ischemia for 45 min, followed by reperfusion for 1 h	10/10/10/10/10/10	A. Control B. Sham C. I/R D. I/R + Zinc E. I/R + Mel F. I/R + Zn + Mel	3 weeks of 3 mg/kg/day *i.p*., before ischemia	/
Yip et al. (2015)	China-Taiwan	Adult male SD rats (325–350 g)	Both ischemia for 1 h, followed by reperfusion	10/10/10/10	A. Sham B. I/R C. I/R + Ex4 D. I/R + Mel E. I/R + Ex4 + Mel	20 mg/kg *i.p*., 30 min post-reperfusion and 50 mg/kg at 6 and 18 h	Scr, BUN, ratio of urine protein to creatinine
Banaei et al. (2016a)	Iran	Male Wistar Albino rats (200–300 g)	Right excised, left ischemia for 45 min, followed by reperfusion for 24 h	10/10/10/10/10	A. Sham B. I/R C. I/R + Mel D. I/R + erythropoietin E. I/R + Mel + erythropoietin	10 mg/kg, *i.p*., 10 min prior to ischemia	Uric acid
Banaei et al. (2016b)	Iran	Male Wistar albino rats (200–300 g)	Right excised, left ischemia for 45 min, followed by reperfusion for 24 h	10/10/10/10	A. Sham B. I/R C. I/R + Mel D. I/R + erythropoietin	10 mg/kg, *i.p*., 10 min prior to ischemia	/
Chang et al. (2016)	China-Taiwan	Pathogen-free, adult male SD rats (320–350 g)	Left ischemia for 1 h, followed by reperfusion	8/8/8/8/8	A. Sham B. I/R C. I/R + Ex4 D. I/R + Mel E. I/R + Ex4 + Mel	20 mg/kg at 0.5 h after IR and 50 mg/kg at 6 and 18 h after I/R	Scr, BUN, ratio of urine protein to creatinine
Shi et al. (2019)	China	Male adult SD rats (250 ± 10 g, 6–8 weeks 7 of age)	Both ischemia for 30 min, then released for 48 h reperfusion	6-8/6-8/6-8/6-8/6-8/6-8	A. Sham B. I/R C. Diabetic + sham D. Diabetic + I/R E. Diabetic + I/R + Mel F. Diabetic + I/R+ Mel + EX5	10 mg/kg, *i.p*., daily for 4 weeks prior to ischemia	BUN, Scr
Souza et al. (2018)	Brazil	Adult male Wistar rats, weighing 276–406 g	Left ischemia for 45 min, followed by reperfusion for 4 h	8/8/8/8	A. I/R B. cold I/R C. Mel+I/R D. Mel+cold I/R	10 mg/kg, *i.p*., 10 min prior to ischemia	Scr, BUN
Chen et al. (2019a)	China	Male SD rats with an age of 7 weeks old, weighing 180–200 g	Both ischemia for 1 h, followed by reperfusion	7/7/7/7/7	A. Sham B. I/R C. I/R + Mel D. I/R+ PAA E. I/R + Mel + PAA	20 mg/kg, *i.p*., at 30 min, 50 mg/kg at 6 h and 18 hr, 50 mg/kg from day 1 to day 7 after reperfusion	/
Chen et al. (2019b)	China	Male SD rats (180–200 g)	Both ischemia for 1 h, followed by reperfusion	7/7/7/7/7	A. Sham B. I/R C. I/R + Mel D. I/R + PAA E. I/R + Mel + PAA	20 mg/kg, *i.p*., at 30 min, 50 mg/kg at 6 h and 18 h after reperfusion	BUN, Scr
M El Agaty and Ibrahim Ahmed (2020)	Egypt	Male Wister rats, weighing 260–280 g	Both ischemia for 45 min, followed by reperfusion	7/7/7	A. Sham B. I/R C. I/R+Mel	15 mg/kg, *p.o*., per day for 2 weeks before IR	BUN, Scr
Wang et al. (2020)	USA	Eight-week-old male C57BL/6 mice	30-min bilateral renal artery ischemia, 24 h or 72 h reperfusion	6/6/6	A. Sham B. I/R C. I/R+Mel	5 mg/kg, *i.p*., 30 min after ischemia	BUN, Scr
Yang et al. (2020)	China	Female C57BL/6 mice	Right ischemia for 40 min, followed by reperfusion	8/8/8/8	A. Sham B. Mel C. I/R + saline D. I/R + Mel	20 mg/kg, *i.p*. 24 and 1 h before ischemia	Scr, BUN
Zahran et al. (2020)	Egypt	Female albino rats	Both ischemia for 40 min, followed by reperfusion	8/8/8/8/8/8/8	A. Control B. Sham C. I/R + saline D. I/R + Mel E. I/R + mesenchymal stem cells F. I/R + exosomes G. I/R + Mel + mesenchymal stem cells H. I/R + Mel + exosomes	20 mg/kg, *i.p*., 3 days after ischemia	BUN, Scr, Retinol-binding protein

*h, hour(s); wk, week(s); I, ischemia; Mel, Melatonin; I/R, ischemia/reperfusion; i.p., intraperitoneally; BUN, blood urea nitrogen; Scr, Serum creatinine; SD, Sprague-Dawley; Px, pinealectomized; min, minute(s); ALT, alanine aminotransferase; AST, aspartate aminotransferase; EPO, erythropoietin; IVR, ischemia-venous blood-reperfusion; LPS, lipopolysaccharide; CrCl, creatinine clearance*.

If any data were only displayed by graphs, the GetData Graph Digitizer 2.24 was adopted to estimate the results. Besides, in order to avoid human error, two reviewers (DRL and LTY) independently extracted relevant data from the papers. If the error was within the acceptable range (error ≤ 1% average data), the average data of two were used. Otherwise, the third investigator (MY) would extract the data again, and take the average of two data which were more close.

### Assessment of the Risk of Bias

The methodological quality was assessed by using the CAMARADES 10-item checklist: (1) peer-reviewed journal; (2) temperature control; (3) animals were randomly allocated; (4) blind established model; (5) blinded outcome assessment; (6) anesthetics used without marked intrinsic neuroprotective properties; (7) animal model (diabetic, advanced age or hypertensive); (8) calculation of sample size; (9) statement of compliance with animal welfare regulations; (10) possible conflicts of interest (Macleod et al., 2004).

Two reviewers (DRL and LTY) assessed the risk of bias. Bias was marked as high or low risk, as well as “unclear” indicated that the risk of bias was unclear. The symbol “+” was used to marked low risk, and it was also recorded as the point of quality score. Agreement over the quality assessment was determined using the Kw. The Kw value >0.75 was accepted. Disagreements were resolved through discussion with the third reviewers (YP).

### Statistical Analysis

If at least three studies reported the results of the same outcome, the data were summarized, thus we evaluated six outcomes separately (BUN, SCr, MDA, MPO, SOD, and GSH). First, we conducted a meta-analysis for studies comparing melatonin group to control group. The Review Manager 5.3 software was adopted to analyzed data. The results in this report were described as weighted mean difference (WMD) of measurements with same unit or standardized mean difference (SMD) using different units. We analyzed the pooled data to evaluate the therapeutic action of melatonin. The heterogeneity of included studies was evaluated by *I*^2^. The fixed effect models were adopted, if the heterogeneity was not obvious (i.e., *p* > 0.1; *I*^2^ ≤ 50%); when *p* ≤ 0.1; *I*^2^ > 50%, the source of heterogeneity was tried to detect by sensitivity analysis. Otherwise, random effect models were used (Higgins and Green, 2011).

Subgroup analysis was conducted between different ischemia duration (≤ 45 min, or > 45 min), times of administration (single or multiple), unilateral or bilateral I/R injury, dosage (< 10, 10, or > 10 mg/kg), administration time (before ischemia, or after reperfusion), and risk of bias (< 4, or ≥ 4). If we include at least 10 studies in a meta-analysis related to primary outcomes, funnel plots were used to test the potential risk of publication bias (Higgins and Green, 2011).

## Results

### Selection of Studies

**Figure 1:**
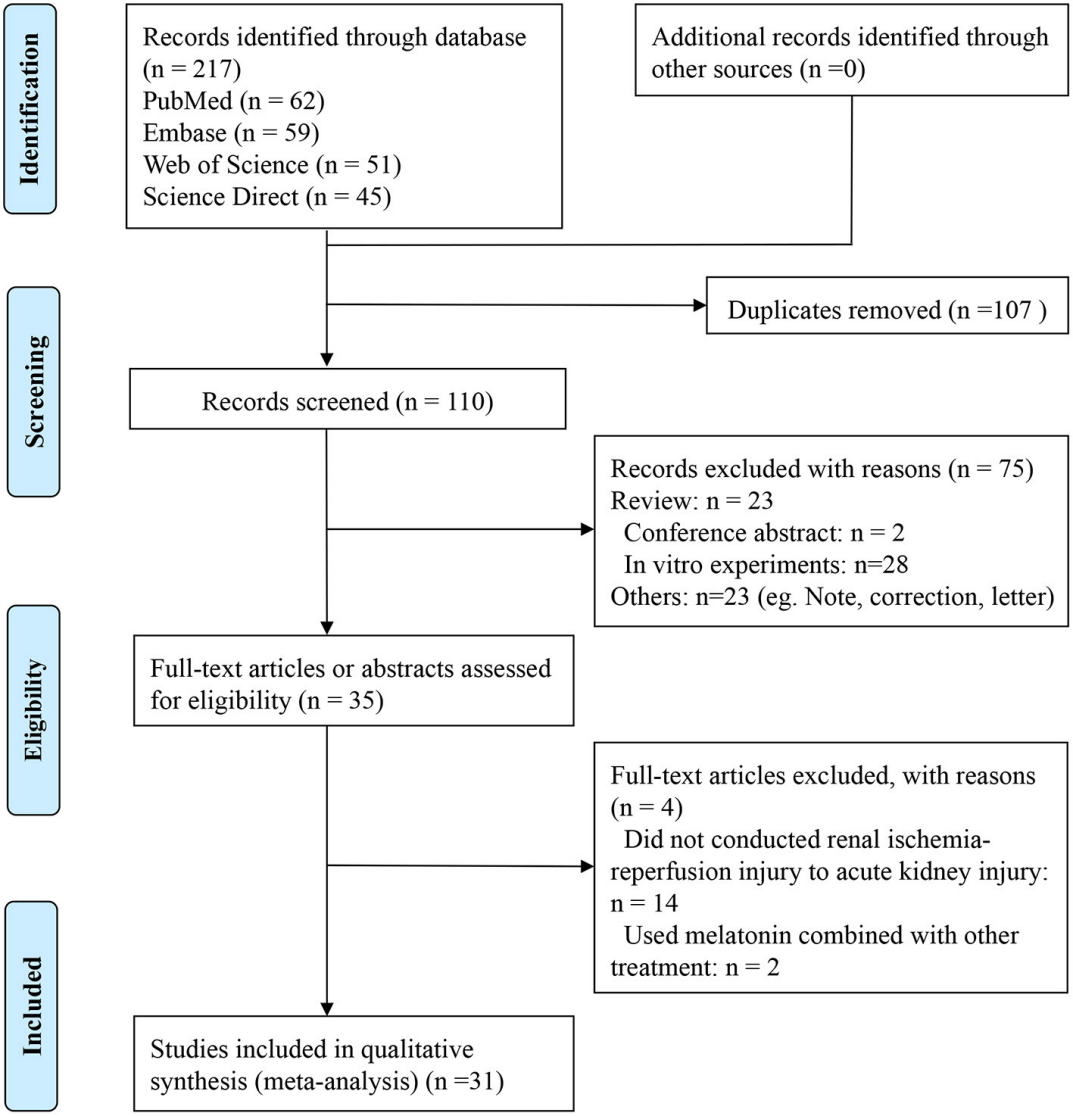
Summary of the literature identification and selection process.

The process of inclusion and exclusion is shown in Figure 1. Two hundred seventeen publications were identified after search. After removing duplicates and performing title and abstract screening, 35 papers were selected for full-text screening, and 31 met the inclusion criteria (Sener et al., 2002; Kunduzova et al., 2003; Sahna et al., 2003; Rodríguez-Reynoso et al., 2004; Aktoz et al., 2007; Kurcer et al., 2007a,b; Fadillioglu et al., 2008; Ersoz et al., 2009; Sinanoglu et al., 2012; Ahmadiasl et al., 2013, 2014a,b; Sezgin et al., 2013; Cetin et al., 2014; Sehajpal et al., 2014; Hadj Ayed Tka et al., 2015; Oguz et al., 2015; Yilmaz et al., 2015; Yip et al., 2015; Banaei et al., 2016a,b; Chang et al., 2016; Souza et al., 2018; Chen et al., 2019a,b; Shi et al., 2019; M El Agaty and Ibrahim Ahmed, 2020; Wang et al., 2020; Yang et al., 2020; Zahran et al., 2020). Of the four publications excluded at the full-text level, two did not conduct renal I/R injury to AKI (Li et al., 2009; Zhu et al., 2017), and the other two used melatonin combined with other treatments (mesenchymal stem cell-derived exosomes, or nitric oxide synthase inhibitor) (Deniz et al., 2006; Alzahrani, 2019). The Kw value of the 2 reviewers was 0.915, after discussion with the third review author, the differences were resolved by consensus.

### Characteristics of the Studies Included

The majority of these studies were conducted in Turkey (*n* = 12), while the remaining in China (*n* = 6), Iran (*n* = 5), Egypt (*n* = 2), Brazil (*n* = 1), USA (*n* = 1), India (*n* = 1), France (*n* = 1), Mexico (*n* = 1), and Tunisia (*n* = 1) (Table 2). A unilateral nephrectomy was performed before contralateral renal I/R injury in 12 studies (Sener et al., 2002; Sahna et al., 2003; Rodríguez-Reynoso et al., 2004; Kurcer et al., 2007a; Sinanoglu et al., 2012; Ahmadiasl et al., 2013, 2014a,b; Sezgin et al., 2013; Oguz et al., 2015; Banaei et al., 2016a,b). Most of the studies conducted unilateral renal I/R injury, except 10 studies performed bilateral renal I/R injury (Ersoz et al., 2009; Sehajpal et al., 2014; Hadj Ayed Tka et al., 2015; Yip et al., 2015; Chen et al., 2019a,b; Shi et al., 2019; M El Agaty and Ibrahim Ahmed, 2020; Wang et al., 2020; Zahran et al., 2020). The duration of ischemia ranged from 25 to 75 min, then the reperfusion from 1 to 72 h. The sample size of a single group ranged between 5 and 12. The melatonin administration was < 10 mg/kg in eight studies (Kunduzova et al., 2003; Sahna et al., 2003; Kurcer et al., 2007b; Fadillioglu et al., 2008; Cetin et al., 2014; Sehajpal et al., 2014; Yilmaz et al., 2015; Wang et al., 2020), 10 mg/kg in 16 studies (Sener et al., 2002; Rodríguez-Reynoso et al., 2004; Aktoz et al., 2007; Kurcer et al., 2007a; Ersoz et al., 2009; Sinanoglu et al., 2012; Ahmadiasl et al., 2013, 2014a,b; Sezgin et al., 2013; Sehajpal et al., 2014; Oguz et al., 2015; Banaei et al., 2016a,b; Souza et al., 2018; Shi et al., 2019), and > 10 mg/kg in eight studies (Hadj Ayed Tka et al., 2015; Yip et al., 2015; Chang et al., 2016; Chen et al., 2019a,b; M El Agaty and Ibrahim Ahmed, 2020; Yang et al., 2020; Zahran et al., 2020). Most studies took melatonin intraperitoneal injection, except one with subcutaneous injection (Sener et al., 2002), one with intravenous injection (Kunduzova et al., 2003), and another one with oral administration (M El Agaty and Ibrahim Ahmed, 2020). Single-dose administration was conducted in 18 studies (Sener et al., 2002; Kunduzova et al., 2003; Sahna et al., 2003; Rodríguez-Reynoso et al., 2004; Kurcer et al., 2007a; Ersoz et al., 2009; Ahmadiasl et al., 2013, 2014a,b; Cetin et al., 2014; Sehajpal et al., 2014; Hadj Ayed Tka et al., 2015; Oguz et al., 2015; Banaei et al., 2016a,b; Souza et al., 2018; Wang et al., 2020; Zahran et al., 2020), while the others used multiple-dose administration (Deniz et al., 2006; Aktoz et al., 2007; Kurcer et al., 2007b; Fadillioglu et al., 2008; Sinanoglu et al., 2012; Sezgin et al., 2013; Yilmaz et al., 2015; Yip et al., 2015; Chang et al., 2016; Chen et al., 2019a,b; Shi et al., 2019; Yang et al., 2020). Two independent reviewers assessed the data, with a Kw of 0.817. Consensus was reached on 100% of the occasions when the reviewers initially disagreed with the third reviewer.

**Table 2:** The research quality of included studies.

**References**	**1**	**2**	**3**	**4**	**5**	**6**	**7**	**8**	**9**	**10**	**Total**
Sener et al. (2002)	+	?	?	?	?	+	+	?	+	?	4
Kunduzova et al. (2003)	+	?	?	?	?	+	+	?	+	+	5
Sahna et al. (2003)	+	+	?	?	+	+	+	?	?	+	6
Rodríguez-Reynoso et al. (2004)	+	+	?	?	?	+	+	?	+	?	5
Aktoz et al. (2007)	+	+	+	?	?	+	+	?	+	?	6
Kurcer et al. (2007a)	+	+	+	?	?	+	+	?	+	+	7
Kurcer et al. (2007b)	+	+	?	?	?	+	+	?	+	+	6
Fadillioglu et al. (2008)	+	+	+	?	?	+	+	?	+	+	7
Ersoz et al. (2009)	+	+	+	?	+	+	+	?	+	?	7
Sinanoglu et al. (2012)	+	?	+	?	+	+	+	?	+	+	7
Ahmadiasl et al. (2013)	+	+	?	?	+	+	+	?	+	?	6
Sezgin et al. (2013)	+	+	+	?	?	+	+	?	+	+	7
Ahmadiasl et al. (2014a)	+	+	?	?	+	+	+	?	+	+	7
Ahmadiasl et al. (2014b)	+	+	?	?	+	+	+	?	+	+	7
Cetin et al. (2014)	+	+	?	?	+	+	+	?	+	+	7
Sehajpal et al. (2014)	+	?	?	?	?	+	+	?	+	?	4
Hadj Ayed Tka et al. (2015)	+	+	?	?	?	+	+	?	+	+	6
Oguz et al. (2015)	+	+	?	?	?	+	+	?	+	+	6
Yilmaz et al. (2015)	+	?	+	?	+	+	+	?	+	?	6
Yip et al. (2015)	+	+	+	?	+	+	+	?	+	?	7
Banaei et al. (2016a)	+	+	?	?	+	+	+	?	+	?	6
Banaei et al. (2016b)	+	+	?	?	+	+	+	?	+	+	7
Chang et al. (2016)	+	+	?	?	+	+	+	?	+	+	7
Shi et al. (2019)	+	+	+	?	+	+	+	?	+	+	8
Souza et al. (2018)	+	+	+	?	+	+	+	?	+	+	8
Chen et al. (2019a)	+	+	+	?	?	+	+	?	+	+	7
Chen et al. (2019b)	+	?	+	?	?	+	+	?	+	+	6
M El Agaty and Ibrahim Ahmed (2020)	+	?	+	?	?	+	+	?	+	+	6
Wang et al. (2020)	+	?	?	?	?	+	+	?	?	?	3
Yang et al. (2020)	+	?	+	?	+	+	+	?	+	+	7
Zahran et al. (2020)	+	?	?	?	?	+	+	?	+	+	5

*Studies fulfilling the criteria of the following: (1) peer reviewed publication; (2) control of temperature; (3) random allocation to treatment or control; (4) blinded induction of model; (5) blinded assessment of outcome; (6) use of anesthetic without significant intrinsic vascular protection activity; (7) appropriate animal model (aged, diabetic, or hypertensive); (8) sample size calculation; (9) compliance with animal welfare regulations; (10) statement of potential conflict of interests*.

### Risk of Bias Within Studies

The Kw value of the 2 reviewers was 0.903, consensus was reached on 100% of the occasions, and the risk of bias was in Table 2. The overall quality of research methodology is good, as 21 (80.65%) out of these 31 studies were labeled as high quality. All of them were peer reviewed publication, and applied appropriate animal model, and anesthetic without significant intrinsic vascular protection activity, but none of them reported sample size calculation, and only 10 studies did not report control of temperature (Sener et al., 2002; Kunduzova et al., 2003; Sinanoglu et al., 2012; Sehajpal et al., 2014; Yilmaz et al., 2015; Chen et al., 2019b; M El Agaty and Ibrahim Ahmed, 2020; Wang et al., 2020; Yang et al., 2020; Zahran et al., 2020). Randomization was conducted in 14 studies (Aktoz et al., 2007; Kurcer et al., 2007b; Fadillioglu et al., 2008; Ersoz et al., 2009; Sinanoglu et al., 2012; Sezgin et al., 2013; Yilmaz et al., 2015; Yip et al., 2015; Souza et al., 2018; Chen et al., 2019a,b; Shi et al., 2019; M El Agaty and Ibrahim Ahmed, 2020; Yang et al., 2020), then no study reported blinded induction of model, and blinded assessment of the outcome was applied in 15 studies (Sahna et al., 2003; Ersoz et al., 2009; Sinanoglu et al., 2012; Ahmadiasl et al., 2013, 2014a,b; Cetin et al., 2014; Yilmaz et al., 2015; Yip et al., 2015; Banaei et al., 2016a; Chang et al., 2016; Souza et al., 2018; Shi et al., 2019; Yang et al., 2020). Most of the studies reported compliance with animal welfare regulations (Sahna et al., 2003; Wang et al., 2020), while 10 studies reported potential conflicts of interest and study funding (Sener et al., 2002; Rodríguez-Reynoso et al., 2004; Aktoz et al., 2007; Ersoz et al., 2009; Ahmadiasl et al., 2013; Sehajpal et al., 2014; Yilmaz et al., 2015; Yip et al., 2015; Banaei et al., 2016a,b; Wang et al., 2020).

### Bun

**Figure 2:**
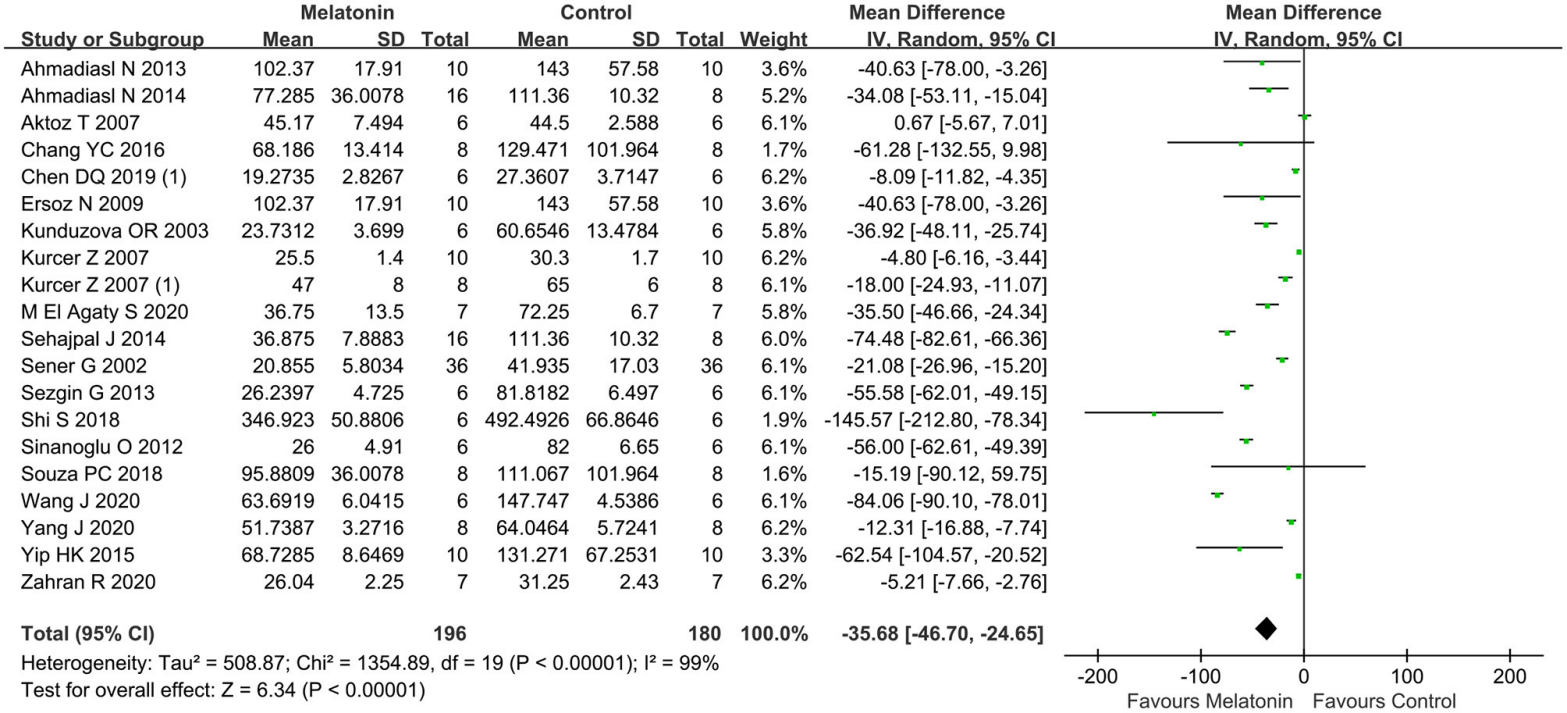
Forest plot for the effects of melatonin on blood urea nitrogen.

The BUN was reported in 21 studies, then animals treated with melatonin showed a greater reduction in BUN level compared with controls (21 studies, *n* = 324; WMD = −30.00; 95% CI = −42.09 to −17.91; *p* < 0.00001; Figure 2), with random-effects model. Subgroup analysis suggested that multiple administration of melatonin could not reduce the BUN level in renal I/R injury model compared with controls (11 studies, *n* = 160; WMD = −26.51; 95% CI = −36.86 to 16.15; *p* < 0.00001; Table 3), while single administration was the opposite (10 studies, *n* = 164; WMD = −28.92; 95% CI = −61.27 to 3.43; *p* = 0.08; Table 3). The other subgroup analysis found that the reduction of BUN level did not differ between different ischemia duration (≤ 45 min, or > 45 min), unilateral or bilateral I/R injury, dosage (< 10, 10, or > 10 mg/kg), administration time (before ischemia, or after reperfusion), or risk of bias (< 4, or ≥ 4). The subgroup analysis also concluded that enhancing the dosage of melatonin did not improve the protective effect of BUN compared to dosage of 10 mg/kg (10 studies, *n* = 148; WMD = −38.07; 95% CI = −70.19 to −5.94; *p* = 0.01; Table 3), and > 10 mg/kg (6 studies, *n* = 92; WMD = −15.25; 95% CI = −22.76 to −7.74; *p* < 0.0001; Table 3).

**Table 3:** Subgroup analysis of pooled estimates of BUN.

**Subgroup**	**No. of studies**	**Sample size**	**WMD (95% CI)**	***P*-value**
**Ischemia duration**				
≤ 45 min	8	65/57	−32.17 [−52.81, −11.53]	0.002
> 45 min	13	105/97	−28.45 [−41.89, −15.01]	< 0.00001
**Unilateral or bilateral**				
Unilateral	13	104/96	−20.49 [−36.03, −4.94]	0.01
Bilateral	8	66/58	−47.06 [−70.78, −23.34]	0.0001
**Dosage**				
< 10 mg/kg	6	44/40	−35.61 [−62.98, −8.23]	0.01
= 10 mg/kg	10	80/68	−38.07 [−70.19, −5.94]	0.02
> 10 mg/kg	6	46/46	−15.25 [−22.76, −7.74]	< 0.0001
**Times of administration**				
Single	10	90/74	−28.92 [−61.27, 3.43]	0.08
Multiple	11	80/80	−26.51 [−36.86, −16.15]	< 0.00001
**Administration time**				
Before ischemia	16	133/117	−27.66 [−42.27, −13.06]	0.0002
After reperfusion	5	37/37	−40.59 [−72.17, −9.01]	< 0.00001
**Risk of bias**				
<4	14	120/104	−24.55 [−38.44, −10.65]	0.0005
≥4	7	50/50	−43.65 [−65.43, −21.69]	< 0.0001

*BUN, blood urea nitrogen; WMD, weighted mean difference; CI, confidence interval*.

### SCr

**Figure 3:**
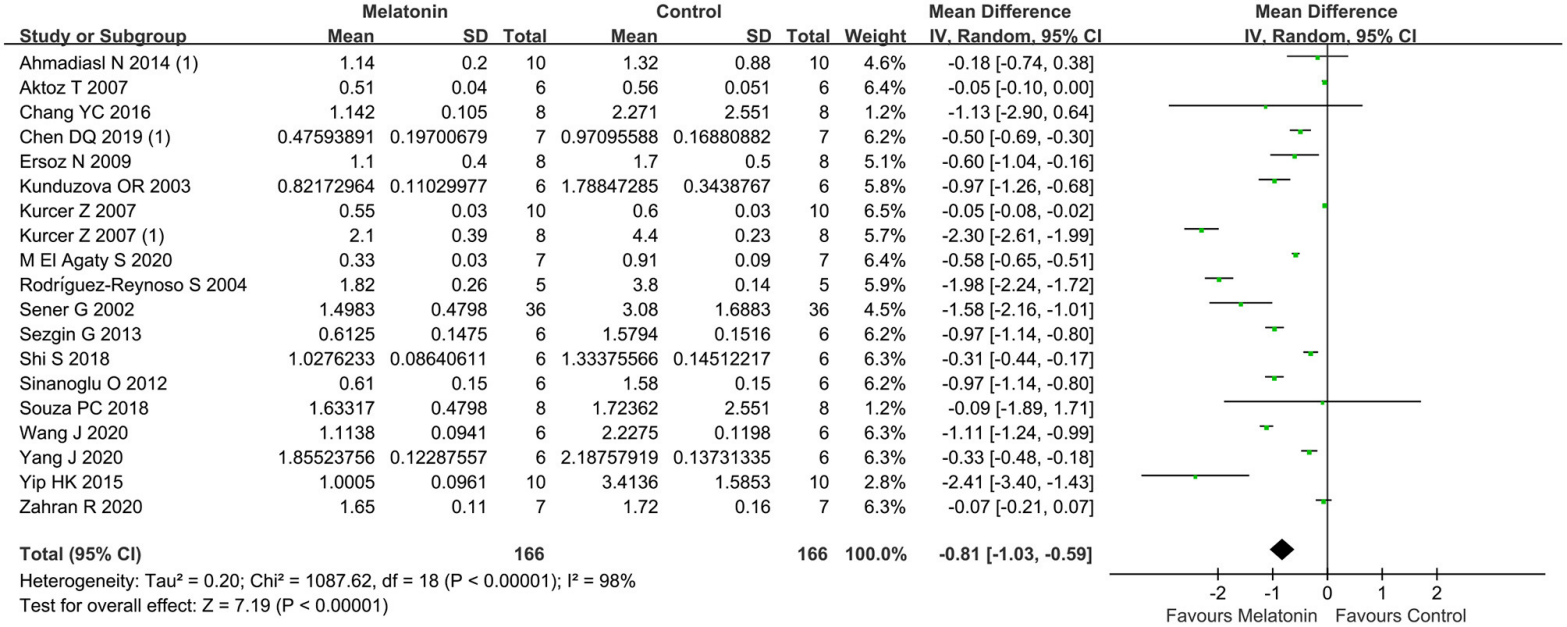
Forest plot for the effects of melatonin on serum creatinine.

The pooled results from 20 studies showed a significant reduction in SCr level with melatonin treatment (20 studies, *n* = 288; WMD = −0.91; 95% CI = −1.17 to −0.66; *p* < 0.00001; Figure 3), with random-effects model. Subgroup analysis suggested that the reduction of SCr level did not differ between different ischemia duration (≤ 45 min, or > 45 min), times of administration (single or multiple), unilateral or bilateral I/R injury, dosage (< 10, 10, or > 10 mg/kg), administration time (before ischemia, or after reperfusion), and risk of bias (< 4, or ≥ 4). The subgroup analysis also concluded that enhancing the dosage of melatonin did not improve the protective effect of SCr compared to dosage of 10 mg/kg (7 studies, *n* = 122; WMD = −1.18; 95% CI = −1.85 to −0.51; *p* = 0.0006; Table 4), and > 10 mg/kg (6 studies, *n* = 94; WMD = −0.57; 95% CI = −0.80 to −0.34; *p* < 0.00001; Table 4).

**Table 4:** Subgroup analysis of pooled estimates of Scr.

**Subgroup**	**No. of studies**	**Sample size**	**WMD (95% CI)**	***P*-value**
**Ischemia duration**				
≤ 45 min	12	86/86	−0.78 [−1.14, −0.41]	< 0.00001
> 45 min	8	58/58	−1.16 [−1.71 −0.61]	< 0.00001
**Unilateral or bilateral**				
Unilateral	13	93/93	−1.04 [−1.39, −0.68]	< 0.00001
Bilateral	7	51/51	−0.64 [−0.94, −0.34]	< 0.00001
**Dosage**				
<10 mg/kg	5	35/35	−0.57 [−1.05, −0.10]	0.02
= 10 mg/kg	7	61/61	−1.18 [−1.85, −0.51]	0.0006
> 10 mg/kg	6	47/47	−0.57 [−0.80, −0.34]	< 0.00001
**Times of administration**				
Single	9	63/63	−1.32 [−1.92, −0.71]	< 0.00001
Multiple	11	81/81	−0.48 [−0.68, −0.29]	< 0.00001
**Administration time**				
Before ischemia	15	106/106	−0.92 [−1.21, −0.63]	< 0.00001
After reperfusion	5	38/38	−0.89 [−1.50, −0.29]	< 0.00001
**Risk of bias**				
<4	13	94/94	−0.21 [−0.23, −0.19]	< 0.00001
≥4	7	50/50	−0.56 [−0.64, −0.49]	< 0.00001

*Scr, Serum creatinine; WMD, weighted mean difference; CI, confidence interval*.

### Outcome Related to Oxidative Stress

**Figure 4:**
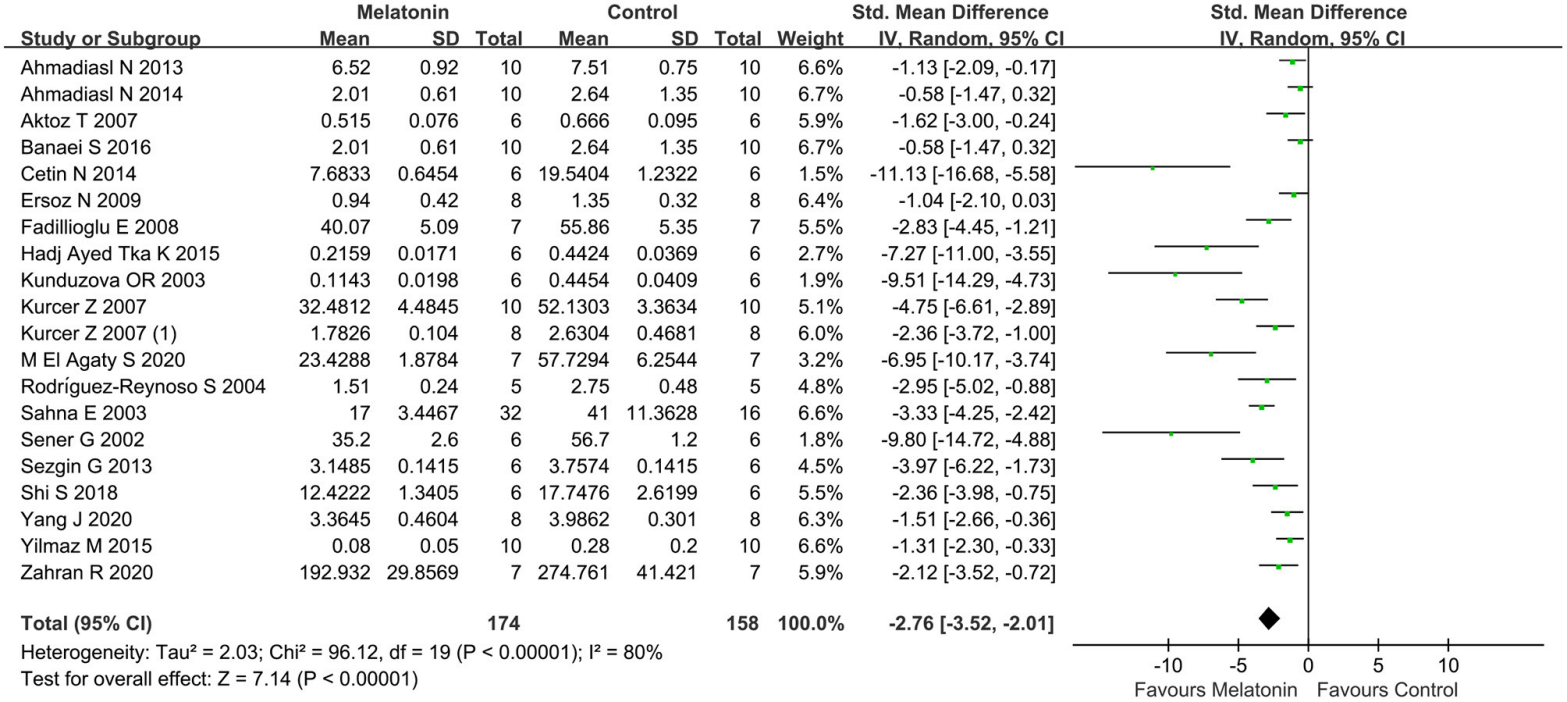
Forest plot for the effects of melatonin on malondialdehyde.

**Figure 5:**
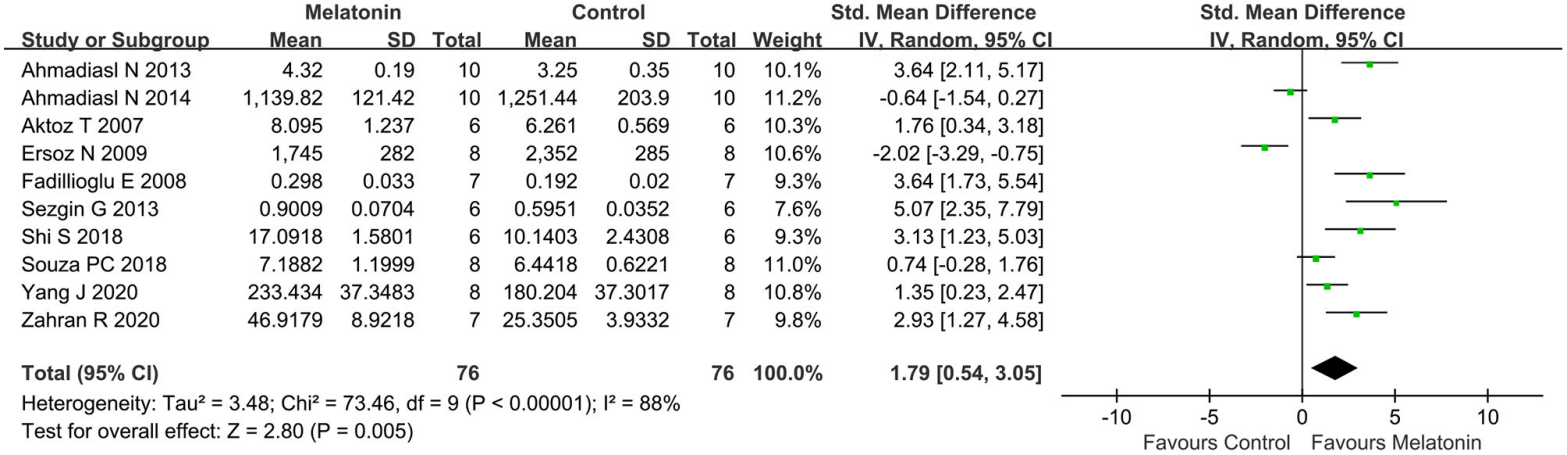
Forest plot for the effects of melatonin on myeloperoxidase.

**Figure 6:**
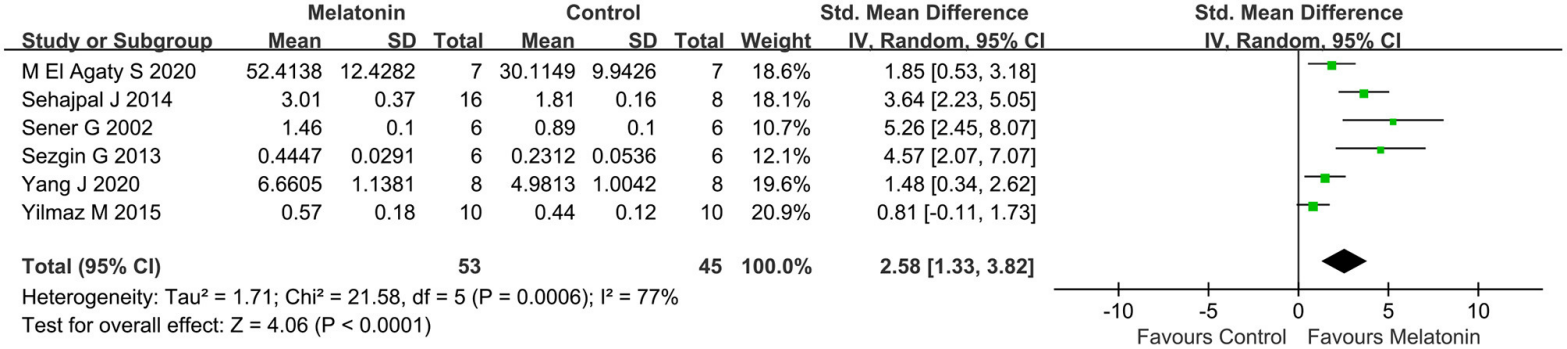
Forest plot for the effects of melatonin on superoxide dismutase.

**Figure 7:**
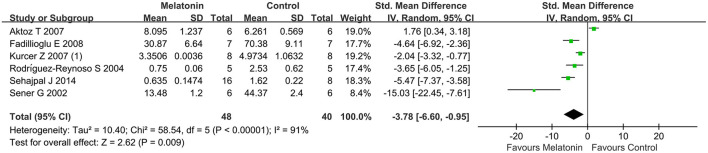
Forest plot for the effects of melatonin on glutathione.

Antioxidative effects were measured in most of the studies, except 9 studies (Kurcer et al., 2007b; Sinanoglu et al., 2012; Oguz et al., 2015; Yilmaz et al., 2015; Banaei et al., 2016b; Chang et al., 2016; Chen et al., 2019a,b; Wang et al., 2020). The MDA level (20 studies, *n* = 332; SMD = −2.76; 95% CI = −3.52 to −2.01; *p* < 0.00001; Figure 4) and the MPO level (6 studies, *n* = 88; SMD = −3.78; 95% CI = −6.60 to −0.95; *p* = 0.009; Figure 5) were significantly reduced after melatonin administration, while the SOD level (10 studies, *n* = 152; SMD = 1.79; 95% CI, 0.54 to 3.05; *P* = 0.005; Figure 6), and GSH level (6 studies, *n* = 98; SMD = 2.58; 95% CI, 1.33–3.82; *P* < 0.0001; Figure 7) were increased compared with control.

### Publication Bias

**Figure 8:**
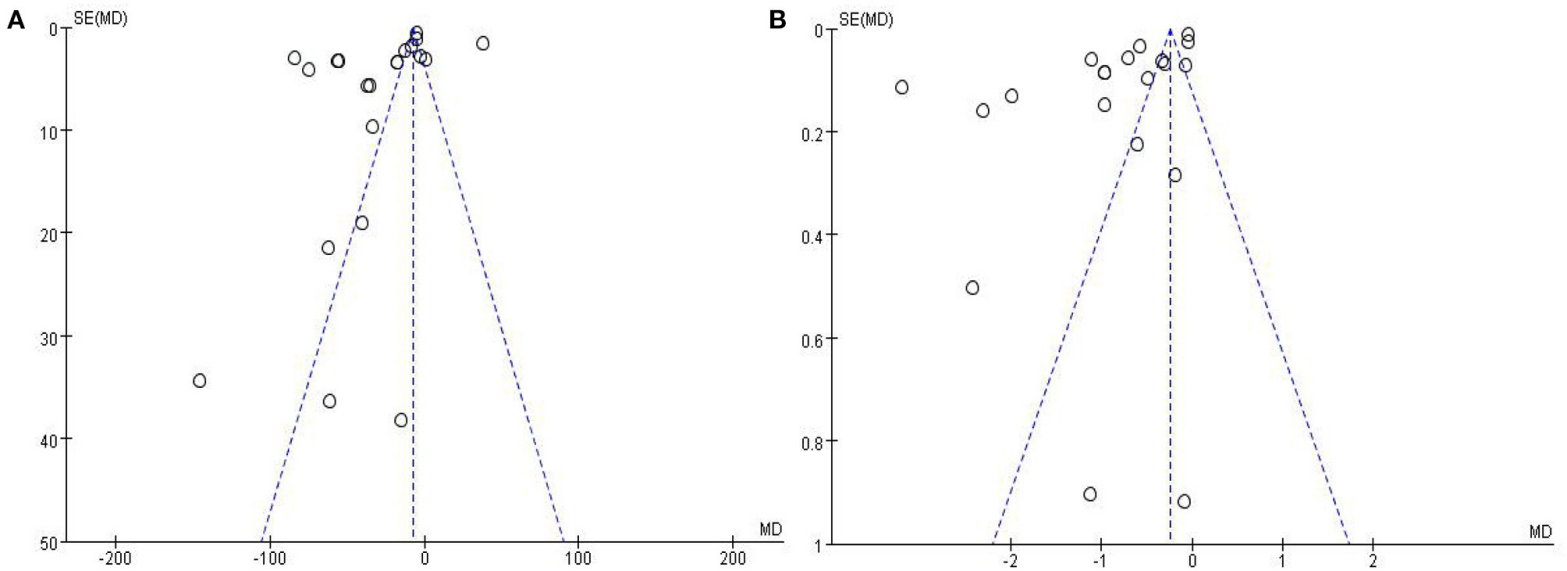
Funnel plots of publication bias for blood urea nitrogen **(A)** and serum creatinine **(B)**.

The funnel plots of BUN and SCr were asymmetrical, hinting at a high risk of publication bias (Figures 8A,B).

## Discussion

### Summary of Evidence

This is the first preclinical meta-analysis of melatonin in the treatment of renal I/R injury. Thirty-one studies compared melatonin to placebo controls were enrolled. The overall quality of all the included studies was good, as there were 21 (80.65%) out of 31 studies were considered high quality.

Our findings indicated that melatonin can significantly improve renal function (BUN and SCr) and reduces oxidative stress (decreasing MDA and MPO, increasing SOD and GSH) of renal post-I/R injury in animals studies. The subgroup analysis also concluded that 10 mg/kg might be the appropriate dosage of melatonin for renal post-I/R injury, while enhancing the dosage of melatonin did not improve the therapeutic action of melatonin for renal I/R injury, and multiple administration might be more effective. Otherwise, the marked benefits of melatonin were not affected by either the duration of ischemia, duration of reperfusion, unilateral or bilateral I/R injury, timing of administration, or methodological quality.

### The Possible Mechanism for the Effect of Melatonin in Renal I/R Injury

**Figure 9:**
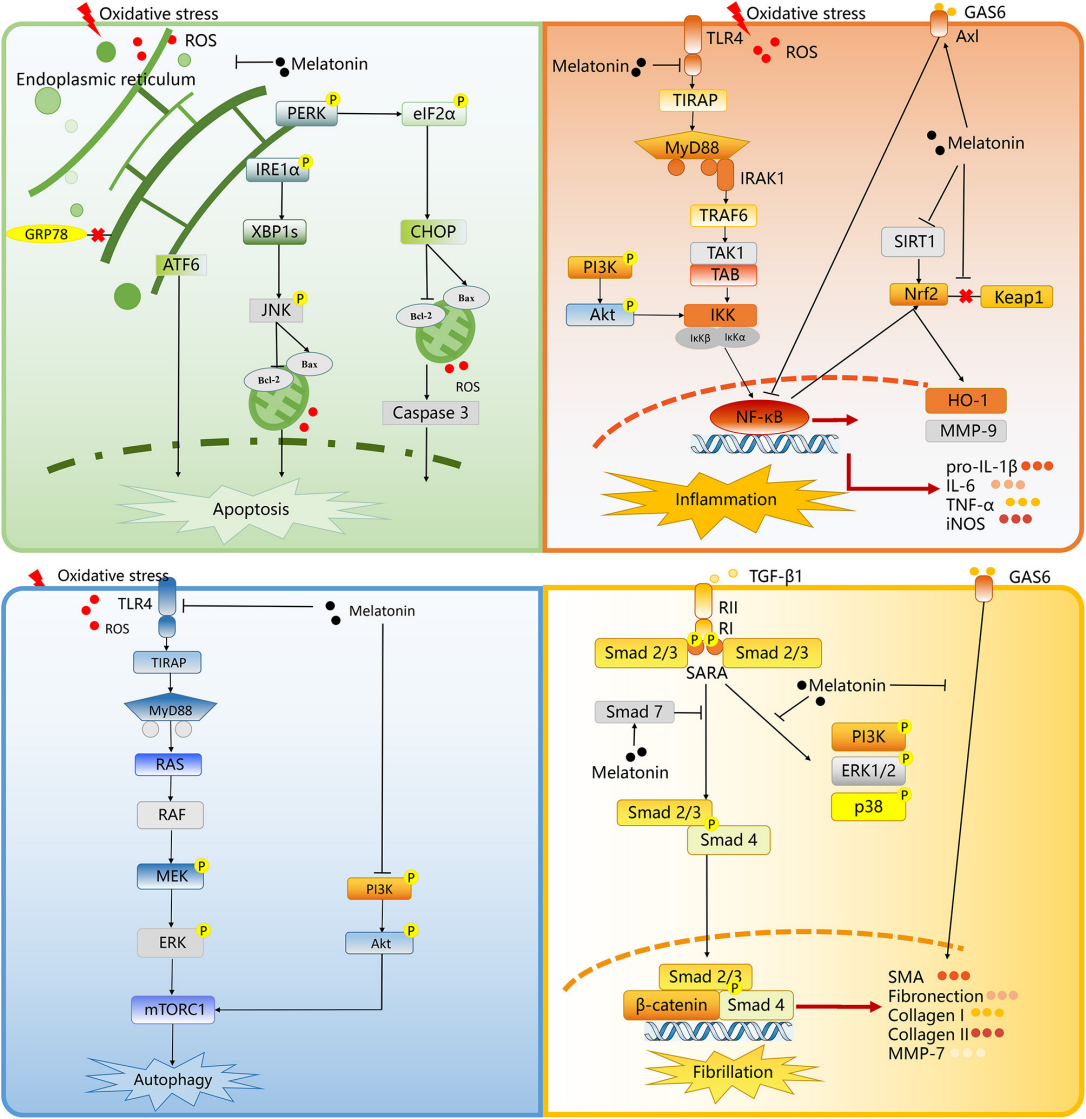
The proposed molecular and cellular mechanism of the protective effect of melatonin for renal I/R injury. The figure was created based on the data of the studies. ROS, reactive oxygen species; PERK, protein kinase RNA-like endoplasmic reticulum kinase; eIF2a, eukaryotic translation initiation factor 2 a; CHOP, C/EBP homologous protein; IRE1a, inositolrequiring enzyme 1 a; XBP1s, X-box binding protein 1; ATF6, activating transcription factor 6; JNK, c-Jun NH2-terminal kinase; Bcl-2, B cell lymphoma-2; Bax, BCL-2 associated X; GRP78, glucose regulated protein 78; TLR4, Toll-like receptor 4; MyD88, myeloid differentiation 88; IRAK1, interleukin-1 receptor-associated kinases; TRAF6, tumor necrosis factor receptor-associated factor 6; TAK1, transforming growth factor beta-activated kinase1; IKK, IkappaB kinase; PI3K, phosphatidylinositol 3-kinase; NF-κB, noncanonical nuclear factor-kappaB; HO-1, heme oxygenase 1; SIRT1, Sirtuin-1; MMP9, matrix metalloproteinase 9; GAS6, growth arrest-specific 6; Keap1, kelch-like ECH-associated protein 1; Nrf2, nuclear factor (erythroid-derived 2) factor 2; IL-6, interleukin 6; pro-IL-1ß, pro-interleukin-1beta; TNF-a, tumor necrosis factor-alpha; iNOS, inducible nitric oxide synthase; RAS, viral oncogene homolog; MEK, mitogen-activated protein kinase; ERK, extracellular regulated protein kinases; mTORC1, mammalian target of rapamycin complex 1; RII, transforming growth factor-ß receptor II; RI, transforming growth factor-ß receptor I; SARA, Smad anchor for receptor activation; TGF-ß1, transforming growth factor-ß; SMA, smooth muscle actin; MMP7, matrix metalloproteinase 7.

At present, there are various mechanisms to explain the origin of tissue damage. An important initiating step in renal I/R injury is the uncontrolled ROS production during reperfusion (Kim et al., 2010). From here, some injury cascades are activated, including loss of endothelial function and programmed leukocyte death (such as apoptosis and autophagy). The activation of the innate and acquired immune system can be achieved through the risk or injury related molecular patterns associated with toll like receptor (TLR) binding and the activation of complement system, resulting in the occurrence of fibrosis associated with chronic transplantation dysfunction (Ben Mosbah et al., 2010). While in this situation, melatonin can function as an antioxidant stress molecule could attenuate apoptosis, autophagy, inflammation, and fibrillation in renal I/R injury (Table 5, Figure 9).

**Table 5:** The proposed molecular and cellular mechanism of the protective effect of melatonin for renal I/R injury.

**References**	**Mechanism**	**Effect**
Sener et al. (2002)	Oxidative stress	Decreased MDA, MPO and PO, increased GSH
Kunduzova et al. (2003)	Oxidative stress, and apoptosis	Decreased MDA, and blocked caspase−3 activity
Sahna et al. (2003)	Oxidative stress	Decreased MDA
Rodríguez-Reynoso et al. (2004)	Oxidative stress, and inflammation	Decreased MDA, MPO, and iNOS, increased GSH, and blocked neutrophil infiltration
Aktoz et al. (2007)	Oxidative stress, cast formation, and tubular necrosis	Decreased MDA, increased SOD, and CAT
Kurcer et al. (2007a)	Oxidative stress	Decreased MDA, PC, and NO
Kurcer et al. (2007b)	Inflammation	Decreased TNF-α, IL-β, and IL-6
Fadillioglu et al. (2008)	Oxidative stress	Decreased MDA, MPO, TAC, and TOS
Ersoz et al. (2009)	Oxidative and nitrosative stress	Decreased MDA, PCC, NOx, SOD, and GSH-Px
Sinanoglu et al. (2012)	Apoptosis	Blocked caspase-3 activity
Ahmadiasl et al. (2013)	Oxidative stress, and inflammation	Decreased MDA, increased SOD, CAT, and GSH-Px, inhibit mononuclear cell infiltration
Sezgin et al. (2013)	Oxidative stress	Decreased MDA and NO, increased SOD, and GSH
Ahmadiasl et al. (2014a)	Oxidative stress	Decreased MDA, increased TAC, SOD, and GSH-Px
Ahmadiasl et al. (2014b)	Oxidative stress, and apoptosis	Decreased MDA and TNF-α, increased TAC, and bcl2
Cetin et al. (2014)	Oxidative stress	Decreased MDA and XO, increased GSH-Px
Sehajpal et al. (2014)	Oxidative stress	Decreased MDA, TBARS and SAG, increased CAT and GSH
Hadj Ayed Tka et al. (2015)	Oxidative stress, ER stress, and apoptosis	Decreased MDA, inhibited ER stress (phosphorylation of GRP 78, p-PERK, ATF 6, CHOP and JNK), and phosphorylation of Akt, GSK-3, VDAC, ERK, and P38
Oguz et al. (2015)	Inflammation	Decreased TNF-α and IL-6
Yilmaz et al. (2015)	Oxidative stress	Decreased MDA, increased GSH
Yip et al. (2015)	Glomerular integrity, Oxidative stress, and Inflammation	Enhanced glomerular integrity (ZO-1, p-cadherin, podocin, dystroglycan, fibronectin), inhibited protein expressions of inflammatory (TNF-α/NF-κB/MMP-9) and oxidative stress (NOX-1, NOX-2, oxidized protein)
Banaei et al. (2016a)	Oxidative stress	Decreased MDA, SOD, and GSH-Px
Banaei et al. (2016b)	Morphological damage	Increase the observed Hb and Hct values, decreased the hyaline cast and thickening of the Bowman capsule basement membrane
Chang et al. (2016)	Inflammation, apoptotic	Inhibited inflammatory (TLR 4, iNOS, and IL-1β), apoptotic (mitochondrial Bax, cleaved caspase-3 and p53), podocyte dysfunction (Wnt1/Wnt4/β-catenin), and enhanced podocyte integrity (E/P-cadherin), and cell survival (PI3K/AKT/mTOR)
Shi et al. (2019)	Oxidative stress, and apoptosis	Decreased MDA, increased SOD, inhibited SIRT1 expression, and Nrf2/HO-1 signaling
Souza et al. (2018)	Oxidative stress	Increased SOD and CAT
Chen et al. (2019a)	Apoptosis, and renal fibrosis	Inhibited the interaction of TGF-β/Smad and Wnt/β-catenin
Chen et al. (2019b)	Oxidative stress and inflammation, fibrosis and podocyte injury	Upregulated Gas6/Axl/NF-κB/Nrf2 signaling to reduce oxidative stress and inflammation in AKI and downregulated Gas6/Axl signaling
M El Agaty and Ibrahim Ahmed (2020)	Oxidative stress	Decreased pancreatic MDA and TNF-α
Wang et al. (2020)	Cytoplasmic calcium overload, myocardial damage, mitochondrial calcium accumulation	Induced phosphorylation of the IP3R/MCU pathways
Yang et al. (2020)	Oxidative stress, apoptotic, inflammation, autophagy	Decreased MDA, TNF-α, IL-2, IL-6, and IL-10 increased SOD, GSH and CAT, inhibited MyD88-dependent TLR4 and MEK/ERK/mTORC1 signaling
Zahran et al. (2020)	Oxidative stress, apoptotic, inflammation	Decreased MDA, IL-1β, kidney injury molecule-1, IL-18, MMP9, TNF-α and NF-κB, increased SOD and CAT, reduced apoptosis (lower DNA damage and bax, and higher bcl-2)

*MDA, malondialdehyde; MPO, myeloperoxidase; PO, protein oxidation; GSH, glutathione; iNOS, inducible nitric oxide synthase; SOD, superoxide dismutase; CAT, catalase; PC, protein carbonyl; NO, nitric oxide; TNF-α, tumor necrosis factor-α; Interleukin-1β, IL-1β; Interleukin-6, IL-6; TAC, total antioxidant capacity; TOS, total oxidative stress; bcl-2, B-cell lymphoma-2; XO, xanthine oxidase; TBARS, thiobarbituric acid reactive substances; SAG, superoxide anion generation; glucose regulated protein 78, GRP 78; p-PERK, phospho-protein kinase R-like endoplasmic reticulum kinase; XBP 1, X-box binding protein 1; ATF-6, activating transcription factor-6; CHOP, C/EBP homoiogousprotein; JNK, c-Jun N-terminal kinase; GSK-3, glycogen synthase kinase 3; VDAC, voltage-dependent anion channels; ERK, extracellular regulated protein kinases; ZO-1, zonula occludens-1; NF-κB, nuclear factor-kappa B; MMP-9, matrix metalloproteinase 9; NOX, nicotinamide adenine dinucleotide phosphate oxidase; Hb, hcthemoglobin; Hct, hematocrit; TLR 4, Toll-like receptor; Nrf2, nuclear factor E2-related factor 2; HO-1, heme oxygenase-1; Inositol 1,4,5-trisphosphate receptor type I, IP3R; MCU, mitochondrial Ca^2+^ uniporter; TGF-β, transforming growth factor-β; growth arrest specific 6, GAS6; MyD88, myeloid differentiation factor 88*.

### Protective Effect of Melatonin Related to Apoptosis

Oxidative stress and other states may cause ER stress. Under the condition of ER stress, ER chaperone, glucose-regulated protein (GRP) 78, leaving from ER membrane, and combining the free misfolding proteins, results in the activation of protein kinase-(PKR-) like ER kinase (PERK), inositol-requiring enzyme 1, and activating transcription factor 6, so as to reconstruct ER homeostasis (Ben Mosbah et al., 2010). Hadj Ayed Tka et al. revealed that melatonin treatment could inhibit ER stress in an experimental model of renal I/R injury (Hadj Ayed Tka et al., 2015). Melatonin inhibits ER stress by suppressing cell apoptosis. Melatonin can play a protective role by inhibiting phosphorylated c-Jun NH2 terminal kinase (JNK), as well as C/EBP homologous protein (CHOP). The CHOP and JNK activation, as downstream events of ER stress, can cause mitochondrial mediated apoptosis by promoting Bax aggregation in mitochondria (Yang et al., 2014; Yan et al., 2018).

### Protective Effect of Melatonin Related to Inflammation

The TLRs act critical roles in the pathogen immune response, and TLR4 is an important regulator related to the inflammatory reaction in I/R injury (Zhang et al., 2016). The myeloid differentiation primary response 88 (MyD88) pathways is the TLR4-induced downstream pathways, then the former pathway promotes the expression of inflammation-associated genes by activating the transcription factors, nuclear factor (NF)-κB. It regulates the expression of a variety of inflammatory factors, such as interleukin (IL)-1β, and IL-6 (Su et al., 2019). Chang et al. and Yang et al. showed that melatonin could down-regulate the pathway of TLR4-MyD88-NF-κB and reduced the deliver of IL-6, IL-1β, as well as tumor necrosis factor (TNF)-α, eventually leading to the reduction of inflammation in the injured area (Chang et al., 2016; Yang et al., 2020). Otherwise, the activation of NF-κB is also enhanced according to growth arrest-specific 6 (Gas6)/Axl. In the early stage of I/R injury, Gas6/Axl inhibits its downstream mediator nuclear factor-erythroid-2-related factor 2 (Nrf2) and NF-κB to attenuate inflammation and oxidative stress after I/R in multiple organs (Llacuna et al., 2010; Ruiz et al., 2013). Oxidative stress and inflammation are closely related, as they jointly construct a vicious circle, that is, oxidative stress causes inflammation, which leading to the recruitment and activation of immunocytes. On the other hand, inflammation promotes oxidative stress by activating leukocytes and resident cells to produce ROS and reactive nitrogen species (He et al., 2017). Chen DQ et al., indicated that melatonin could upregulate Gas6/Axl to down-regulate NF-κB/Nrf2 signaling to reduce oxidative stress, as well as inflammation in the renal I/R injury early stage (Chen et al., 2019b).

### Protective Effect of Melatonin Related to Autophagy

Autophagy plays a cardinal contribution in renal I/R injury. Injurious autophagy may lead to the release of excessive ROS and pro apoptotic factors, which ultimately cause cell death (Kurusu and Kuchitsu, 2017; Liu et al., 2019). Yang et al. suggested that a major pathway for melatonin-induced autophagy in I/R injury involves activating the TLR4-MyD88 signaling pathway (Yang et al., 2020). Mitogen-activated protein kinases (MAPK) pathways is actived by the MyD88 pathway (Liu et al., 2018). Mammalian target of rapamycin complex 1 (mTORC1), a convergence point for many upstream pathways, including MAPKs, regulates autophagy. Yang et al. revealed that melatonin-induced autophagy among renal I/R mice was dependent on downregulation of mTORC1 by its upstream activator, the TLR4/MyD88 (Yang et al., 2020).

Otherwise, it is known that phosphatidylinositol-3-kinase (PI3K)/AKT/mTOR pathway perform a core effect to regulate cell survival, as well as cell growth, and proliferation (Hassan et al., 2013). Another important finding of Chang YC was that PI3K/AKT/mTORC1 signaling pathway was significantly upregulated in the I/R kidney, while melatonin can significantly inhibit this signal pathway (Chang et al., 2016). It was claimed that the activation of PI3K/AKT/mTOR signaling under the I/R condition is the internal response of tissue or organs to I/R (i.e., the more serious, the stonger response), and melatonin could reverse this situation (Chang et al., 2016).

### Protective Effect of Melatonin Related to Fibrillation

Fibrillation plays a leading role in renal I/R injury to accelerate kidney damage from AKI to CKD condition. The Wnt/β-catenin, as well as transforming growth factor-β (TGF-β)/Smad pathways perform important effects in the fibrosis of CKD (Yang et al., 2010; Meng et al., 2015). Under damage conditions, Wnt/β-catenin signaling is activated (Chen et al., 2017; Wang et al., 2017), and its sustained activation can promote renal fibrosis to induce the transition from AKI to CKD (Xiao et al., 2016). Fibrotic, as well as profibrotic genes, including twist, snail1, fibroblast-specific protein 1, and plasminogen activator inhibitor-1, are the downstream molecules related to Wnt/β-catenin signaling. TGF-β1 phosphorylate Smad2 and Smad3 to activate receptors through the combination with its adaptor, Smad anchor with its receptors TGF-β receptor I and II (Wang et al., 2017). The phosphorylated Smad2 and Smad3 pass through Wnt/β-catenin signaling to regulate the expression the profibrotic factor. Smad4 is also critical to TGF-β1-mediated fibrogenesis (Morishita et al., 2014), while Smad7, as the negative regulation factor of the TGF-β/Smad pathway, could inhibit phosphorylate Smad2 and Smad3 (Meng et al., 2012). Chen et al. demonstrated that melatonin performed extensive inhibitory effects on the TGF-β/Smad and Wnt/β-catenin pathways to prevent the AKI from progressing to CKD (Chen et al., 2019b).

Otherwise, in AKI to CKD condition, Gas6/Axl mentioned earlier serves as a profibrotic route and accelerates fibrogenesis (Bárcena et al., 2015). Then, melatonin could downregulate Gas6/Axl signaling pathway to prevent the fibrosis of the kidney (Chen et al., 2019b).

### Advantage and Limitation of This Review

Combining the advantages of systematic review and traditional review, the study pooled the previous data to show that melatonin is a promising drug for renal I/R injury, and summarized the mechanism of melatonin. A deeper understanding of the mechanism of melatonin to treat renal I/R injury would not only help to explore the pathological mechanisms during renal I/R injury, but also contribute to the investigation of new effective drugs for further development and related translational research.

Inevitably, the article also has some limitations. First, since we cannot obtain the data of individual animals, the study can only be meta-analyzed at the overall level of each study (based on mean and standard deviation). The renal I/R injury models used of each study are different, different ischemia times for instance. The simple hybrid method is not the most appropriate method, but we can't analyze all the inconsistencies hierarchically. Then, the funnel chart is asymmetric, so the potential publication deviation cannot be ignored, which may affect the accuracy of the results.

## Conclusion

This systematic review demonstrated that melatonin could improve renal function and antioxidative effects in renal I/R injury from the available data of small animal studies. A possible mechanism is that it can reduce ROS, thereby inhibiting apoptosis, inflammation, autophagy, and finally stopping fibrillation in AKI to CKD situation. Melatonin could be a promising drug to treat renal I/R injury. Nonetheless, extensive animal experimental studies are need to explore the mechanism of melatonin, and well-designed randomized controlled trials to explore the protective effect of melatonin.

## Data Availability Statement

The original contributions presented in the study are included in the article/supplementary material, further inquiries can be directed to the corresponding author/s.

## Author Contributions

The study was conceived by Rl-D, G-cQ, and YP. R-lD, T-yL, JT, X-hH, J-mM, Y-qS, and W-jZ developed the eligibility criteria, search strategy, risk of bias assessment strategy, and data extraction plan with guidance from G-cQ and YP. Rl-D wrote the manuscript. All authors contributed to the article and approved the submitted version.

## Funding

This work was funded by National Natural Science Foundation of China (No. 81904070), Leading talents plan of TCM of Shanghai (No. ZY-2018-2020 - RCPY - 1017), and supported by Shanghai Key TCM specialty training program.

## Conflict of Interest

The authors declare that the research was conducted in the absence of any commercial or financial relationships that could be construed as a potential conflict of interest.

## Publisher's Note

All claims expressed in this article are solely those of the authors and do not necessarily represent those of their affiliated organizations, or those of the publisher, the editors and the reviewers. Any product that may be evaluated in this article, or claim that may be made by its manufacturer, is not guaranteed or endorsed by the publisher.

## References

[B1] Ahmadiasl N. Banaei S. Alihemati A. Baradaran B. Azimian E. (2014a). Effect of a combined treatment with erythropoietin and melatonin on renal ischemia reperfusion injury in male rats. Clin. Exp. Nephrol. 18, 855–864. 10.1007/s10157-014-0937-6 24493464

[B2] Ahmadiasl N. Banaei S. Alihemmati A. (2013). Combination antioxidant effect of erythropoietin and melatonin on renal ischemia-reperfusion injury in rats. Iran. J. Basic Med. Sci. 16, 1209–1216 24570825 PMC3933796

[B3] Ahmadiasl N. Banaei S. Alihemmati A. Baradaran B. Azimian E. (2014b). The anti-inflammatory effect of erythropoietin and melatonin on renal ischemia reperfusion injury in male rats. Adv. Pharm. Bull. 4, 49–54. 10.5681/apb.2014.008 24409409 PMC3885368

[B4] Aktoz T. Aydogdu N. Alagol B. Yalcin O. Huseyinova G. Atakan I. H. (2007). The protective effects of melatonin and vitamin E against renal ischemia-reperfusion injury in rats. Ren. Fail. 29, 535–542. 10.1080/08860220701391738 17654314

[B5] Alzahrani F. A. (2019). Melatonin improves therapeutic potential of mesenchymal stem cells-derived exosomes against renal ischemia-reperfusion injury in rats. Am. J. Transl. Res. 11, 2887–2907. 31217862 PMC6556638

[B6] Banaei S. Ahmadiasl N. Alihemmati A. (2016a). Combination anti-apoptotic effect of erythropoietin and melatonin on ischemia reperfusion-induced renal injury in rats. Acta Med. Iran. 54, 624–630. 27888589

[B7] Banaei S. Ahmadiasl N. Alihemmati A. (2016b). Comparison of the protective effects of erythropoietin and melatonin on renal ischemia-reperfusion injury. Trauma Mon. 21:e23005. 10.5812/traumamon.23005 27921018 PMC5124127

[B8] Bansal N. Matheny M. E. Greevy R. A. Jr. Eden S. K. Perkins A. M. Parr S. K. . (2018). Acute kidney injury and risk of incident heart failure among US veterans. Am. J. Kidney Dis. 71, 236–245. 10.1053/j.ajkd.2017.08.027 29162339

[B9] Bárcena C. Stefanovic M. Tutusaus A. Joannas L. Menéndez A. García-Ruiz C. . (2015). Gas6/Axl pathway is activated in chronic liver disease and its targeting reduces fibrosis via hepatic stellate cell inactivation. J. Hepatol. 63, 670–678. 10.1016/j.jhep.2015.04.013 25908269 PMC4543529

[B10] Basile D. P. Donohoe D. Roethe K. Osborn J. L. (2001). Renal ischemic injury results in permanent damage to peritubular capillaries and influences long-term function. Am. J. Physiol. Renal Physiol. 281, F887–899. 10.1152/ajprenal.00050.2001 11592947

[B11] Ben Mosbah I. Alfany-Fernández I. Martel C. Zaouali M. A. Bintanel-Morcillo M. Rimola A. . (2010). Endoplasmic reticulum stress inhibition protects steatotic and non-steatotic livers in partial hepatectomy under ischemia-reperfusion. Cell Death Dis. 1:e52. 10.1038/cddis.2010.29 21364657 PMC3032561

[B12] Carrascal L. Nunez-Abades P. Ayala A. Cano M. (2018). Role of melatonin in the inflammatory process and its therapeutic potential. Curr. Pharm. Des. 24, 1563–1588. 10.2174/1381612824666180426112832 29701146

[B13] Cetin N. Suleyman H. Sener E. Demirci E. Gundogdu C. Akcay F. (2014). The prevention of ischemia/reperfusion induced oxidative damage by venous blood in rabbit kidneys monitored with biochemical, histopatological and immunohistochemical analysis. J. Physiol. Pharmacol. 65, 383–392. 24930510

[B14] Chang Y. C. Hsu S. Y. Yang C. C. Sung P. H. Chen Y. L. Huang T. H. . (2016). Enhanced protection against renal ischemia-reperfusion injury with combined melatonin and exendin-4 in a rodent model. Exp. Biol. Med. 241, 1588–1602. 10.1177/1535370216642528 27037275 PMC4994899

[B15] Chawla L. S. Kimmel P. L. (2012). Acute kidney injury and chronic kidney disease: an integrated clinical syndrome. Kidney Int. 82, 516–524. 10.1038/ki.2012.208 22673882

[B16] Chen D. Q. Cao G. Chen H. Liu D. Su W. Yu X. Y. . (2017). Gene and protein expressions and metabolomics exhibit activated redox signaling and wnt/β-catenin pathway are associated with metabolite dysfunction in patients with chronic kidney disease. Redox Biol. 12, 505–521. 10.1016/j.redox.2017.03.017 28343144 PMC5369369

[B17] Chen D. Q. Cao G. Zhao H. Chen L. Yang T. Wang M. . (2019a). Combined melatonin and poricoic acid A inhibits renal fibrosis through modulating the interaction of Smad3 and β-catenin pathway in AKI-to-CKD continuum. Ther. Adv. Chronic Dis. 10:2040622319869116. 10.1177/2040622319869116 31452866 PMC6696851

[B18] Chen D. Q. Feng Y. L. Chen L. Liu J. R. Wang M. Vaziri N. D. . (2019b). Poricoic acid A enhances melatonin inhibition of AKI-to-CKD transition by regulating Gas6/AxlNFκB/Nrf2 axis. Free Radic. Biol. Med. 134, 484–497. 10.1016/j.freeradbiomed.2019.01.046 30716432

[B19] Deniz E. Colakoglu N. Sari A. Sonmez M. F. Tugrul I. Oktar S. . (2006). Melatonin attenuates renal ischemia-reperfusion injury in nitric oxide synthase inhibited rats. Acta Histochem. 108, 303–309. 10.1016/j.acthis.2006.04.002 16764913

[B20] Ersoz N. Guven A. Cayci T. Uysal B. Turk E. Oztas E. . (2009). Comparison of the efficacy of melatonin and 1400W on renal ischemia/reperfusion injury: a role for inhibiting iNOS. Ren. Fail. 31, 704–710. 10.3109/08860220903085989 19814638

[B21] Fadillioglu E. Kurcer Z. Parlakpinar H. Iraz M. Gursul C. (2008). Melatonin treatment against remote organ injury induced by renal ischemia reperfusion injury in diabetes mellitus. Arch. Pharm. Res. 31, 705–712. 10.1007/s12272-001-1216-3 18563351

[B22] Fuquay R. Renner B. Kulik L. McCullough J. W. Amura C. Strassheim D. . (2013). Renal ischemia-reperfusion injury amplifies the humoral immune response. J. Am. Soc. Nephrol. 24, 1063–1072. 10.1681/ASN.2012060560 23641055 PMC3699821

[B23] Hadj Ayed Tka K. Mahfoudh Boussaid A. Zaouali M. A. Kammoun R. Bejaoui M. Ghoul Mazgar S. . (2015). Melatonin modulates endoplasmic reticulum stress and Akt/GSK3-beta signaling pathway in a rat model of renal warm ischemia reperfusion. Anal. Cell. Pathol. 2015:635172. 10.1155/2015/635172 26229743 PMC4502281

[B24] Hassan B. Akcakanat A. Holder A. M. Meric-Bernstam F. (2013). Targeting the PI3-kinase/Akt/mTOR signaling pathway. Surg. Oncol. Clin. N. Am. 22, 641–664. 10.1016/j.soc.2013.06.008 24012393 PMC3811932

[B25] He L. Wei Q. Liu J. Yi M. Liu Y. Liu H. . (2017). AKI on CKD: heightened injury, suppressed repair, and the underlying mechanisms. Kidney Int. 92, 1071–1083. 10.1016/j.kint.2017.06.030 28890325 PMC5683166

[B26] Higgins J. P. T. Green S. E. (2011). Cochrane Handbook for Systematic Reviews of Interventions Version 5.1.0 [updated March 2011]. The Cochrane Collaboration. Available online at: https://training.cochrane.org/handbook

[B27] Inagi R. (2009). Endoplasmic reticulum stress in the kidney as a novel mediator of kidney injury. Nephron Exp. Nephrol. 112, e1–9. 10.1159/000210573 19342868

[B28] Kim J. Jang H. S. Park K. M. (2010). Reactive oxygen species generated by renal ischemia and reperfusion trigger protection against subsequent renal ischemia and reperfusion injury in mice. Am. J. Physiol. Renal Physiol. 298, F158–166. 10.1152/ajprenal.00474.2009 19864300

[B29] Kunduzova O. R. Escourrou G. Seguelas M. H. Delagrange P. De La Farge F. Cambon C. . (2003). Prevention of apoptotic and necrotic cell death, caspase-3 activation, and renal dysfunction by melatonin after ischemia/reperfusion. FASEB J. 17, 872–874. 10.1096/fj.02-0504fje 12670883

[B30] Kurcer Z. Oguz E. Ozbilge H. Baba F. Aksoy N. Celik H. . (2007a). Melatonin protects from ischemia/reperfusion-induced renal injury in rats: this effect is not mediated by proinflammatory cytokines. J. Pineal Res. 43, 172–178. 10.1111/j.1600-079X.2007.00459.x 17645695

[B31] Kurcer Z. Parlakpinar H. Vardi N. Tasdemir S. Iraz M. Fadillioglu E. (2007b) Protective effects of chronic melatonin treatment against renal ischemia/reperfusion injury in streptozotocin-induced diabetic rats. Exp. Clin. Endocrinol. diabet. 115, 365–371. 10.1055/s-2007-971056. 17701881

[B32] Kurusu T. Kuchitsu K. (2017). Autophagy, programmed cell death and reactive oxygen species in sexual reproduction in plants. J. Plant Res. 130, 491–499. 10.1007/s10265-017-0934-4 28364377

[B33] Levey A. S. James M. T. (2017). Acute kidney injury. Ann. Internal Med. 167, Itc66–itc80. 10.7326/AITC201711070 29114754

[B34] Li Z. Nickkholgh A. Yi X. Bruns H. Gross M. L. Hoffmann K. . (2009). Melatonin protects kidney grafts from ischemia/reperfusion injury through inhibition of NF-kB and apoptosis after experimental kidney transplantation. J. Pineal Res. 46, 365–372. 10.1111/j.1600-079X.2009.00672.x 19552759

[B35] Liu H. Dong J. Song S. Zhao Y. Wang J. Fu Z. . (2019). Spermidine ameliorates liver ischaemia-reperfusion injury through the regulation of autophagy by the AMPK-mTOR-ULK1 signalling pathway. Biochem. Biophys. Res. Commun. 519, 227–233. 10.1016/j.bbrc.2019.08.162 31493865

[B36] Liu H. Zhou K. Liao L. Zhang T. Yang M. Sun C. (2018). Lipoxin A4 receptor agonist BML-111 induces autophagy in alveolar macrophages and protects from acute lung injury by activating MAPK signaling. Respir. Res. 19:243. 10.1186/s12931-018-0937-2 30518355 PMC6282312

[B37] Llacuna L. Bárcena C. Bellido-Martín L. Fernández L. Stefanovic M. Marí M. . (2010). Growth arrest-specific protein 6 is hepatoprotective against murine ischemia/reperfusion injury. Hepatology. 52, 1371–1379. 10.1002/hep.23833 20730776 PMC2947564

[B38] M El Agaty S. Ibrahim Ahmed A (2020). Pathophysiological and immunohistochemical analysis of pancreas after renal ischemia/reperfusion injury: protective role of melatonin. Arch. Physiol. Biochem. 126, 264–275. 10.1080/13813455.2018.1517182 30270672

[B39] Macleod M. R. O'Collins T. Howells D. W. Donnan G. A. (2004). Pooling of animal experimental data reveals influence of study design and publication bias. Stroke 35, 1203–1208. 10.1161/01.STR.0000125719.25853.20 15060322

[B40] Meng X. M. Huang X. R. Xiao J. Chung A. C. Qin W. Chen H. Y. . (2012). Disruption of Smad4 impairs TGF-β/Smad3 and Smad7 transcriptional regulation during renal inflammation and fibrosis *in vivo* and *in vitro*. Kidney Int. 81, 266–279. 10.1038/ki.2011.327 22048127

[B41] Meng X. M. Tang P. M. Li J. Lan H. Y. (2015). TGF-β/Smad signaling in renal fibrosis. Front. Physiol. 6:82. 10.3389/fphys.2015.00082 25852569 PMC4365692

[B42] Morishita Y. Yoshizawa H. Watanabe M. Ishibashi K. Muto S. Kusano E. . (2014). siRNAs targeted to Smad4 prevent renal fibrosis *in vivo*. Sci. Rep. 4:6424. 10.1038/srep06424 25236771 PMC4168270

[B43] Mortezaee K. Najafi M. Farhood B. Ahmadi A. Potes Y. Shabeeb D. . (2019). Modulation of apoptosis by melatonin for improving cancer treatment efficiency: an updated review. Life Sci. 228, 228–241. 10.1016/j.lfs.2019.05.009 31077716

[B44] Oguz E. Yilmaz Z. Ozbilge H. Baba F. Tabur S. Yerer M. B. . (2015). Effects of melatonin on the serum levels of pro-inflammatory cytokines and tissue injury after renal ischemia reperfusion in rats. Ren. Fail. 37, 318–322. 10.3109/0886022X.2014.991263 25519208

[B45] Panah F. Ghorbanihaghjo A. Argani H. Haiaty S. Rashtchizadeh N. Hosseini L. . (2019). The effect of oral melatonin on renal ischemia-reperfusion injury in transplant patients: a double-blind, randomized controlled trial. Transpl. Immunol. 57:101241. 10.1016/j.trim.2019.101241 31446153

[B46] Patel S. Rahmani B. Gandhi J. Seyam O. Joshi G. Reid I. . (2020). Revisiting the pineal gland: a review of calcification, masses, precocious puberty, and melatonin functions. Int. J. Neurosci. 130, 464–475. 10.1080/00207454.2019.1692838 31714865

[B47] Reiter R. J. Tan D. X. Manchester L. C. Qi W. (2001). Biochemical reactivity of melatonin with reactive oxygen and nitrogen species: a review of the evidence. Cell Biochem. Biophys. 34, 237–256. 10.1385/CBB:34:2:237 11898866

[B48] Rodríguez-Reynoso S. Leal C. Portilla-de Buen E. Castillo J. C. Ramos-Solano F. (2004). Melatonin ameliorates renal ischemia/reperfusion injury. J. Surg. Res. 116, 242–247. 10.1016/j.jss.2003.10.002 15013362

[B49] Ruiz S. Pergola P. E. Zager R. A. Vaziri N. D. (2013). Targeting the transcription factor Nrf2 to ameliorate oxidative stress and inflammation in chronic kidney disease. Kidney Int. 83, 1029–1041. 10.1038/ki.2012.439 23325084 PMC3633725

[B50] Sahna E. Parlakpinar H. Ozturk F. Cigremis Y. Acet A. (2003). The protective effects of physiological and pharmacological concentrations of melatonin on renal ischemia-reperfusion injury in rats. Urol. Res. 31, 188–193. 10.1007/s00240-003-0314-5 12719947

[B51] Sehajpal J. Kaur T. Bhatti R. Singh A. P. (2014). Role of progesterone in melatonin-mediated protection against acute kidney injury. J. Surg. Res. 191, 441–447. 10.1016/j.jss.2014.04.025 24878191

[B52] Sener G. Sehirli A. O. Keyer-Uysal M. Arbak S. Ersoy Y. Yegen B. C. (2002). The protective effect of melatonin on renal ischemia-reperfusion injury in the rat. J. Pineal Res. 32, 120–126. 10.1034/j.1600-079x.2002.1848.x 12071469

[B53] Sezgin G. Oztürk G. Güney S. Sinanoglu O. Tunçdemir M. (2013). Protective effect of melatonin and 1,25-dihydroxyvitamin D3 on renal ischemia-reperfusion injury in rats. Ren. Fail. 35, 374–379. 10.3109/0886022X.2012.760409 23356461

[B54] Shi S. Lei S. Tang C. (2019). Melatonin attenuates acute kidney ischemia/reperfusion injury in diabetic rats by activation of the SIRT1/Nrf2/HO-1 signaling pathway. Biosci. Rep. 39:BSR20181614. 10.1042/BSR20181614 30578379 PMC6331666

[B55] Sinanoglu O. Sezgin G. Ozturk G. Tuncdemir M. Guney S. Aksungar F. B. . (2012). Melatonin with 1,25-dihydroxyvitamin D3 protects against apoptotic ischemia-reperfusion injury in the rat kidney. Ren. Fail. 34, 1021–1026. 10.3109/0886022X.2012.700887 22780560

[B56] Smith S. F. Hosgood S. A. Nicholson M. L. (2019). Ischemia-reperfusion injury in renal transplantation: 3 key signaling pathways in tubular epithelial cells. Kidney Int. 95, 50–56. 10.1016/j.kint.2018.10.009 30606429

[B57] Souza P. C. Santos E. B. D. Motta G. L. Bona S. R. Schaefer P. G. Campagnol D. . (2018). Combined effects of melatonin and topical hypothermia on renal ischemia-reperfusion injury in rats. Acta Cirurgica Brasileira 33, 197–206. 10.1590/s0102-865020180030000001 29668777

[B58] Su S. Zhang P. Zhang Q. Yin Z. (2019). GSK-3β inhibitor induces expression of the TLR4/MyD88/NF-κB signaling pathway to protect against renal ischemia-reperfusion injury during rat kidney transplantation. Inflammation 42, 2105–2118. 10.1007/s10753-019-01074-2 31440938

[B59] Wang J. Toan S. Li R. Zhou H. (2020). Melatonin fine-tunes intracellular calcium signals and eliminates myocardial damage through the IP3R/MCU pathways in cardiorenal syndrome type 3. Biochem. Pharmacol. 174:113832. 10.1016/j.bcp.2020.113832 32006470

[B60] Wang M. Chen D. Q. Wang M. C. Chen H. Chen L. Liu D. . (2017). Poricoic acid ZA, a novel RAS inhibitor, attenuates tubulo-interstitial fibrosis and podocyte injury by inhibiting TGF-β/Smad signaling pathway. Phytomedicine 36, 243–253. 10.1016/j.phymed.2017.10.008 29157821

[B61] Webster A. C. Nagler E. V. Morton R. L. Masson P. (2017). Chronic kidney disease. Lancet 389, 1238–1252. 10.1016/S0140-6736(16)32064-5 27887750

[B62] Xia Y. Chen S. Zeng S. Zhao Y. Zhu C. Deng B. . (2019). Melatonin in macrophage biology: current understanding and future perspectives. J. Pineal Res. 66:e12547. 10.1111/jpi.12547 30597604

[B63] Xiao L. Zhou D. Tan R. J. Fu H. Zhou L. Hou F. F. . (2016). Sustained activation of Wnt/β-Catenin signaling drives AKI to CKD progression. J. Am. Soc. Nephrol. 27, 1727–1740. 10.1681/ASN.2015040449 26453613 PMC4884114

[B64] Yan M. Shu S. Guo C. Tang C. Dong Z. (2018). Endoplasmic reticulum stress in ischemic and nephrotoxic acute kidney injury. Ann. Med. 50, 381–390. 10.1080/07853890.2018.1489142 29895209 PMC6333465

[B65] Yang F. Huang X. R. Chung A. C. Hou C. C. Lai K. N. Lan H. Y. (2010). Essential role for Smad3 in angiotensin II-induced tubular epithelial-mesenchymal transition. J. Pathol. 221, 390–401. 10.1002/path.2721 20593491

[B66] Yang J. Liu H. Han S. Fu Z. Wang J. Chen Y. . (2020). Melatonin pretreatment alleviates renal ischemia-reperfusion injury by promoting autophagic flux via TLR4/MyD88/MEK/ERK/mTORC1 signaling. FASEB J. 34, 12324–12337. 10.1096/fj.202001252R 32662527

[B67] Yang J. R. Yao F. H. Zhang J. G. Ji Z. Y. Li K. L. Zhan J. . (2014). Ischemia-reperfusion induces renal tubule pyroptosis via the CHOP-caspase-11 pathway. Am. J. Physiol. Renal Physiol. 306, F75–84. 10.1152/ajprenal.00117.2013 24133119

[B68] Yilmaz M. Mogulkoc R. Baltaci A. K. (2015). Effect of three-week zinc and melatonin supplementation on the oxidant-antioxidant system in experimental renal ischemia-reperfusion in rats. Acta Clin. Croat. 54, 395–401. 27017711

[B69] Yip H. K. Yang C. C. Chen K. H. Huang T. H. Chen Y. L. Zhen Y. Y. . (2015). Combined melatonin and exendin-4 therapy preserves renal ultrastructural integrity after ischemia-reperfusion injury in the male rat. J. Pineal Res. 59, 434–447. 10.1111/jpi.12273 26309060

[B70] Zahran R. Ghozy A. Elkholy S. S. El-Taweel F. El-Magd M. A. (2020). Combination therapy with melatonin, stem cells and extracellular vesicles is effective in limiting renal ischemia-reperfusion injury in a rat model. Int. J. Urol. 27, 1039–1049. 10.1111/iju.14345 32794300

[B71] Zhang H. M. Zhang Y. (2014). Melatonin: a well-documented antioxidant with conditional pro-oxidant actions. J. Pineal Res. 57, 131–146. 10.1111/jpi.12162 25060102

[B72] Zhang J. Xia J. Zhang Y. Xiao F. Wang J. Gao H. . (2016). HMGB1-TLR4 signaling participates in renal ischemia reperfusion injury and could be attenuated by dexamethasone-mediated inhibition of the ERK/NF-κB pathway. Am. J. Transl. Res. 8, 4054–4067. 27829992 PMC5095301

[B73] Zhu F. Chong Lee Shin O. L. Xu H. Zhao Z. Pei G. Hu Z. . (2017). Melatonin promoted renal regeneration in folic acid-induced acute kidney injury via inhibiting nucleocytoplasmic translocation of HMGB1 in tubular epithelial cells. Am. J. Transl. Res. 9, 1694–1707. 10.1093/ndt/gfx142.SP182 28469775 PMC5411918

